# Nanopore sequencing data and structural variants identified in *Prunus avium* seedlings derived through mutagenesis

**DOI:** 10.1016/j.dib.2022.108384

**Published:** 2022-06-22

**Authors:** Per McCord, Seanna Hewitt, Amit Dhingra

**Affiliations:** a Washington State University, Irrigated Agriculture Research and Extension Center, Prosser, WA 99350, USA; b Department of Horticulture, Washington State University, Pullman, WA 99164, USA; c Department of Horticultural Sciences, Texas A&M University, College Station, TX 77845, USA

**Keywords:** Mutation breeding, Long-read sequencing, *Prunus avium*, Rosaceae, Structural genomics

## Abstract

DNA from four sweet cherry seedlings derived from gamma-irradiated female parents was sequenced via nanopore technology (Oxford Nanopore MinION). Total data yield was 8.07 Gb, ranging from 0.92 to 3.36 Gb per sample, with the average length of mapped reads ranging from 22 Kbp–24 Kbp. Sequence data was then analysed to identify and characterize variants using a published sweet cherry reference genome. Small and medium-sized indels (55–135 bp), as well as structural variants, including several large indels and complex variants were detected. Of these, 20 variants were localized within protein-coding gene sequences, including those encoding a putative F-box protein, an ADP-ribose glyxohydrolase protein, a predicted 26S protease regulatory subunit, an E3 ubiquitin protein ligase, a UDP-galactose/UDP-blucose transporter, an alpha/beta hydrolase domain-containing protein, a rhodanese-like domain-containing protein, a cytochrome p450 protein, phosphoinositide phosphatase, cysteine synthase-like, phosphoenolpyruvate carboxylase 4, and several uncharacterized proteins. These variations could have functional and phenotypic consequences that are useful in basic research and breeding.

## Specifications Table

**Table:** 

Subject	Biological Science: Omics: General
Specific subject area	Structural genomics and mutation breeding of tree fruit crops
Type of data	Tables containing information regarding the raw sequencing data, mapping data, indel calls, and structural variant calls. Supplementary File 1 containing Excel versions of all manuscript tables. Supplementary File 2 containing sequencing QC reports for each sample.
How the data were acquired	DNA sequence data were acquired via nanopore sequencing (Oxford Nanopore MinION flow cells and MinKNOW basecalling software). Variant call data was acquired using CLC Genomics Workbench (version 21.0.5, https://digitalinsights.qiagen.com/).
Data format	Raw (FastQ sequence data) Analyzed Filtered
Description of data collection	Factors under study included four sweet cherry seedlings derived from irradiated female parents and anonymous (open-pollinated) male parents. Genomic DNA was sequenced from each seedling.
Data source location	• *Institution:* Washington State University • *City/Town/Region:* Prosser, WA and Pullman, WA • *Country:* United States of America
Data accessibility	Repository name: NCBI SRA Database (raw sequence reads) Data identification numbers: BioProject: PRJNA761776; SRA Accessions: SRR15825585; SRR15825584; SRR15825583; SRR15825582 Direct URL to data: https://www.ncbi.nlm.nih.gov/bioproject/PRJNA761776 Repository name: Mendeley Data (FASTA files of indel and structural variant sequences) Direct URL to data: http://dx.doi.org/10.17632/bd5xhv99n8.1

## Value of the Data

• Mutation breeding can be used to introduce novel traits such as self-compatibility and dwarfing.
• Irradiation commonly introduces large scale lesions in DNA, including chromosomal rearrangements and large deletions.
• Breeders and geneticists working on sweet cherry (or related *Prunus* species) can benefit from this data.
• These data can be used to guide targeted phenotyping experiments (including proteomics/metabolomics) to characterize the effects of the mutations identified and to develop markers to track the mutations in progeny for breeding or research purposes.

## 1 Data Description

Historically, mutation breeding has been used in sweet cherry to introduce novel traits such as self-compatibility and dwarfing [1,2]. Long-read DNA sequencing technologies, such as nanopore sequencing, are ideally suited for the detection of large-scale changes to DNA structure. The data presented herein include the raw nanopore sequencing data referenced in “Data accessibility” above. In addition, four tables and two supplementary data files are included. Table 1 is a summary of the total number of reads (sequences) and the total number of nucleotides sequenced for each of the four sweet cherry samples. Table 2 lists the percentage of raw sequence data that was mapped to the reference sweet cherry genome, and the average length of both mapped and un-mapped reads. A list of the short (up to 135 bases) insertions detected in the sequence analysis are shown in Table 3, and a description of the larger structural variants is included in Table 4. Tables 3 and 4 also include any predicted genes affected by such variants. Supplementary File 1 contains all manuscript tables in Excel format. Supplementary File 2 contains QC reports for sequencing reads for each sample. The structural variants (from Table 4) are first, followed by the short insertions. Collectively, these data are useful in demonstrating the utility of nanopore sequencing for genome characterization in sweet cherry, and the variations identified herein are a foundation for additional research in functional genetics and breeding.

**Table 1:** Total number of raw reads and total number of nucleotides sequenced for each cherry sample.

	Raw Data		
	# Reads	# Bases (Data)	Average Read Length
**Cherry 1–15**	55,843	1,227,327,051 (1.23 Gb)	21,978
**Cherry 2–2**	40,421	918,137,204 (0.92 Gb)	22,714
**Cherry 3–1**	143,470	3,362,441,292 (3.36 Gb)	23,437
**Cherry 3–14**	109,846	2,563,398,969 (2.56 Gb)	23,336
**Total**	**349,580**	**8.07 Gb**	**22,866**

**Table 2:** Read mapping statistics for each cherry sample.

	Read Mapping Data												
	# Reference Seqs	# Reference Bases	Total Read Count	# Reads Mapped	% Reads Mapped	Average Length of Mapped Reads	# Mapped Bases	% Mapped Bases	# Unmapped Reads	% Unmapped Reads	Average Length of Unmapped Reads	# Unmapped Bases	% Unmapped Bases
**Cherry 1-15**	9	373,751,615	55,843	54,922	98.35	22,328	1,22,62,82,225	99.91	921	1.65	1134	1044,826	0.09
**Cherry 2-2**			40,421	39,888	98.68	23,006	91,76,61,094	99.95	533	1.32	893	476,110	0.05
**Cherry 3-1**			1,43,470	1,42,524	99.34	23,582	3,36,09,53,360	99.96	946	0.66	1573	1487,932	0.04
**Cherry 3-14**			1,09,846	1,08,861	99.10	23,537	2,56,22,57,696	99.96	985	0.90	1159	1141,273	0.04

**Table 3:** List of short and medium-sized indels identified for each sample, their genomic location, length, supporting evidence, and genes containing variant breakpoints.

	Chromosome	Region	Type	Length	Zygosity	Evidence	Variant ratio	# Variant Reads	Sequence complexity	Gene ID	Gene Annotation
**Cherry 1-15**	PAV_r1.0chr5	16977406^16977407	Insertion	135	Homozygous	Tandem duplication	1	6	0.785149016		
	PAV_r1.0chr7	4543893^4543894	Insertion	87	Homozygous	Tandem duplication	1	2	0.42738503		
	PAV_r1.0chr7	6368305^6368306	Insertion	118	Homozygous	Tandem duplication	1	2	0.172357694		

**Cherry 2-2**	PAV_r1.0chr4	17053980^17053981	Insertion	91	Homozygous	Tandem duplication	1	2	0.436136937	Pav_sc0000326.1_g170.1.mk	PREDICTED putative F-box protein At3g17480
	PAV_r1.0chr7	19712901^19712902	Insertion	63	Homozygous	Tandem duplication	1	2	0.573000973	Pav_sc0000557.1_g210.1.mk	PREDICTED poly(ADP-ribose) glycohydrolase 1-like

**Cherry 3-1**	PAV_r1.0chr1	35284480^35284481	Insertion	55	Homozygous	Tandem duplication	1	2	0.190844691

**Table 4:** List of structural variants identified for each sample, their genomic location, supporting evidence, and genes containing variant breakpoints.

	Chromosome	Region	Type	Evidence	Length	Variant ratio	# Variant Reads	Sequence complexity	Gene ID	Gene Annotation
**Cherry 1-15**	PAV_r1.0chr1	1069112..38245179	Insertion	Tandem duplication	37,176,068	1	2	0.09	Pav_sc0000449.1_g160.1.mk; Pav_sc0000257.1_g250.1.mk	None assigned; PREDICTED protein FAM91A1
	PAV_r1.0chr2	11124496..12408370	Insertion	Tandem duplication	1,283,875	1	5	0.34		
	PAV_r1.0chr2	11124503..12408370	Insertion	Tandem duplication	1,283,868	1	2	0.35		
	PAV_r1.0chr2	11124634..12408370	Inversion	Cross mapped breakpoints	1,283,737	1	5	0.14		
	PAV_r1.0chr3	20603906..22159320	Deletion	Cross mapped breakpoints	1,555,415	1	2	0.32	Pav_sc0001080.1_g310.1.mk	PREDICTED RING finger protein 10 isoform X1
	PAV_r1.0chr3	415334..20603909	Complex	Cross mapped breakpoints (invalid orientation)	20,188,576	1	2	0.44		
	PAV_r1.0chr4	14391180..24139652	Insertion	Tandem duplication	9,748,473	1	2	0.21		
	PAV_r1.0chr4	17053821..21929340	Insertion	Tandem duplication	4,875,520	1	3	0.31	Pav_sc0000326.1_g170.1.mk	PREDICTED: putative F-box protein At3g17480
	PAV_r1.0chr4	749804..15575609	Inversion	Cross mapped breakpoints	14,825,806	1	2	0.31	Pav_sc0000824.1_g170.1.mk; Pav_sc0000218.1_g140.1.mk	PREDICTED 26S protease regulatory subunit 10B homolog A; PREDICTED AP-5 complex subunit zeta-1

**Cherry 2-2**	PAV_r1.0chr2	11124505..23262036	Insertion	Tandem duplication	12,137,532	1	3	0.66	Pav_sc0001405.1_g740.1.mk	PREDICTED LOW QUALITY PROTEIN E3 ubiquitin-protein ligase XBAT33
	PAV_r1.0chr8	3624014…13680696	Complex	Multiple breakpoints	10,056,683	1.5	6	0.47		

**Cherry 3-1**	PAV_r1.0chr2	8437203..14284511	Deletion	Cross mapped breakpoints	5,847,309	1	2	0.38	Pav_sc0001673.1_g150.1.mk	PREDICTED UDP-galactose/UDP-glucose transporter 2-like
	PAV_r1.0chr3	142091..11694992	Complex	Multiple breakpoints	11,552,902	1.5	4	0.15	Pav_sc0001124.1_g370.1.mk	hypothetical protein PRUPE_ppa006355mg
	PAV_r1.0chr3	11694991^11694992	Insertion	Paired breakpoint	2704	1	3	0.29		
	PAV_r1.0chr4	6187635..9039997	Complex	Cross mapped breakpoints (invalid orientation)	2,852,363	1	2	0.31	Pav_sc0000600.1_g170.1.mk	PREDICTED alpha/beta hydrolase domain-containing protein 17B
	PAV_r1.0chr4	21804710..21929340	Complex	Multiple breakpoints	124,631	1.5	7	0.14		
	PAV_r1.0chr5	11874508..12445540	Deletion	Cross mapped breakpoints	571,033	1	2	0.28	Pav_sc0000063.1_g120.1.mk; Pav_sc0000229.1_g410.1.mk	PREDICTED uncharacterized protein LOC103338047; PREDICTED rhodanese-like domain-containing protein 11, chloroplastic; cytochrome P450 71AP13
	PAV_r1.0chr7	7442466..14768544	Inversion	Cross mapped breakpoints	7,326,079	1	2	0.31	Pav_sc0000825.1_g230.1.br; Pav_sc0000414.1_g200.1.mk	hypothetical protein VITISV_007508; PREDICTED phosphoinositide phosphatase SAC6

**Cherry 3-14**	PAV_r1.0chr1	6273648..23662569	Insertion	Tandem duplication	17,388,922	1	2	0.37	Pav_sc0000065.1_g500.1.mk; Pav_sc0000588.1_g830.1.mk	PREDICTED cysteine synthase-like; PREDICTED phosphoenolpyruvate carboxylase 4
	PAV_r1.0chr2	6379117..11124487	Insertion	Tandem duplication	4,745,371	1	2	0.29		
	PAV_r1.0chr4	21929335..24851307	Inversion	Cross mapped breakpoints	2,921,973	1	4	0.43		
	PAV_r1.0chr5	8040277..13000570	Complex	Cross mapped breakpoints (invalid orientation)	4960,295	1	2	0.30	Pav_sc0001309.1_g1020.1.br	hypothetical protein PRUPE_ppa026535mg, partial

**Supplementary Data File 1**. Excel workbook containing Tables 1–4.

**Supplementary Data File 2.** QC reports for sequencing reads for each sample.

## 2 Experimental Design, Materials and Methods

### 2.1 Plant Material

The plant material consisted of seedling progeny of irradiated sweet cherry varieties ‘Royal Ann’, ‘Bada’, and ‘Bing’. Irradiation was accomplished by placing newly sprouted shoots of each variety in a radiation chamber with a ^60^Co gamma ray source. Following irradiation, the shoots were immediately grafted onto a rootstock for propagation. Mutant shoots with reduced or compact growth were repropagated by budding (a form of grafting using single buds). When the mutants proved unstable (likely due to chimerism), open-pollinated seed from the mutant trees was collected and planted, and the less vigorous seedlings were selected and propagated vegetatively via budding/grafting. A planting of 12 selections (vegetatively propagated seedling progeny), each with three replicates, was established at the Oregon State University Mid-Columbia Agricultural Research and Extension Center in Hood River, OR. Of the 12 selections, four were sequenced: 1-15, 2-2, 3-1, and 3-14.

### 2.2 DNA Extraction and Nanopore Sequencing

Tissue from field-grown newly expanded leaves was ground to a fine powder in liquid nitrogen using a mortar and pestle. DNA was extracted using a CTAB-based buffer, washed with 70% ethanol, and the dried pellet was re-suspended in low EDTA buffer (10 mM Tris, 0.1 mmn EDTA, pH 8.0). The DNA was quantified using a NanoDrop spectrophotometer and diluted to a concentration of 150 ng/µL. Prior to sequencing, DNA fragments <25 Kb were removed using a Circulomics Short Read Eliminator Kit [3]. A total of 9 µg of DNA (the maximum for the SRE kit) was processed for each sample according to manufacturer instructions and re-suspended in 50 µL of the provided elution buffer. DNA repair, end-prep, native barcode ligation (for multiplexing), and adapter ligation/cleanup were performed using reagents supplied and/or recommended by Oxford Nanopore Technologies (ONT) with the exceptions that Agencourt AMPure XP beads were replaced with custom made beads (2% v/v Speed Beads, 18% w/v PEG-8000, 1M NaCl, 100 mM Tris pH 8.0, 1 mM EDTA pH 8.0), and gently flicking the tubes every 60–120 seconds instead of using a rotator mixer. Samples were pooled prior to loading on the MinION flow cell. Two samples were barcoded and sequenced per flow cell for a total of four samples (1-15, 2-2, 3-1, and 3-14). The flow cell was then loaded into a MinION DNA sequencer attached to a desktop computer. Sequence data (acquisition and basecalling) was collected from the MinION for 72 h using MinKnow software v. 19.12.5. The raw sequencing read files were uploaded to the NCBI SRA database (BioProject: PRJNA761776).

### 2.3 Sequence Analysis

#### 2.3.1 Read Processing

A summary of raw sequencing reads for each cherry sample is shown in Table 1 and Supplementary File 1. Sequencing quality assessment was performed using the CLC Genomics Workbench ‘QC for sequencing reads’ tool (Supplementary File 2) Reads were mapped to the *Prunus avium* reference genome [4,5] using CLC's “Map Long Reads to Reference (beta) [Long Read Support 21.0]” tool (CLC Genomics Workbench 20.0.5, CLC Long Read Support 21.0 (https://digitalinsights.qiagen.com/). The following parameters were used: Enable long-read spliced alignment = No; Match score = 2; Mismatch cost = 4; Gap open cost = 4; Gap extend cost = 2; Long gap open cost = 24; Long gap extend cost = 1. Mapping results are shown in Table 2.

Structural variants, indels, and putative chromosomal breakpoints were identified using CLC's “Indels and Structural Variants” tool with the following parameters: *P*-Value threshold = 0.001, Maximum number of mismatches = 3, Minimum quality score = 20; Minimum relative consensus coverage = 0.5, Filter variants = Yes; Minimum number of reads = 2; Ignore broken pairs = No, Create breakpoints = Yes, Create Indel variants = Yes, Create structural variations = Yes. A detailed report containing positional location of all identified variants was also generated. The data were additionally filtered for variants, indels, and breakpoints present in genes, and the resulting selections extracted. The final number of SVs and Indels for each genotype that passed the specified filtering parameters is as follows: 1-15 – 9 structural variants, 3 Indels; 2-2 – 2 structural variants, 2 Indels; 3-1 – 7 structural variants, 1 Indel; 3–14 – 4 structural variants, 0 Indels (Table 3, Table 4, Supplementary File 1).

#### 2.3.2 Annotation with Overlap Information

The .gff file containing the gene annotation information corresponding to the *Prunus avium* reference genome pseudomolecule (v1.0.a1) was imported into CLC to generate Gene, Exon, and CDS tracks [6]. To identify which of the putative variant end breakpoints were associated in coding regions of the sweet cherry genome, the CLC “Annotate with Overlap Information” feature was used to add the information from the imported gene tracks to the called variant datasets for each genotype. Gene ID and annotation information for indels and structural variants is shown in Tables 3 and 4.

## CRediT authorship contribution statement

**Per McCord:** Conceptualization, Investigation, Writing – original draft. **Seanna Hewitt:** Formal analysis, Writing – original draft, Data curation. **Amit Dhingra:** Supervision, Writing – review & editing.

## Declaration of Competing Interest

The authors declare that they have no known competing financial interests or personal relationships that could have appeared to influence the work reported in this paper.

## Data Availability

Structural Variant Detection in Four Sweet Cherry F1s Derived from Irradiated Parents (Original data) (NCBI SRA Database).

## Appendix Supplementary materials

Image, application 1

Image, application 2
