# Supplementary material for: Nanopore sequencing data and structural variants identified in Prunus avium seedlings derived through mutagenesis
Source: Data Brief. 2022 Jun 22;43:108384. doi: 10.1016/j.dib.2022.108384 (PMC9253453; doi:10.1016/j.dib.2022.108384)
Supplement: Supplementary file 2 [file mmc2.pdf]

Sequencing QC Report
Based upon: 55,843 sequences in 1 data set
Generated by: root
Creation date: Mon Nov 22 09:55:51 CET 2021
Software: CLC Genomics Workbench 21.0.4

Table of contents

1. Summary 3
2. Per-sequence analysis 3
    2.1 Lengths distribution 3
    2.2 GC-content 4
    2.3 Ambiguous base-content 4
    2.4 Quality distribution 5
3. Per-base analysis 5
    3.1 Coverage 6
    3.2 Nucleotide contributions 6
    3.3 GC-content 7
    3.4 Ambiguous base-content 7
    3.5 Quality distribution 8
4. Over-representation analyses 8
    4.1 Enriched 5-mers 9
    4.2 Sequence duplication levels 9
    4.3 Duplicated sequences 10

1. Summary

Creation date:	Mon Nov 22 09:55:51 CET 2021
Generated by:	root
Software:	CLC Genomics Workbench 21.0.4
Based upon:	1 data set
Cherry_1-15:	55,843 sequences
Total sequences in data set	55,843 sequences
Total nucleotides in data set	1,227,327,051 nucleotides

2. Per-sequence analysis

2.1 Lengths distribution

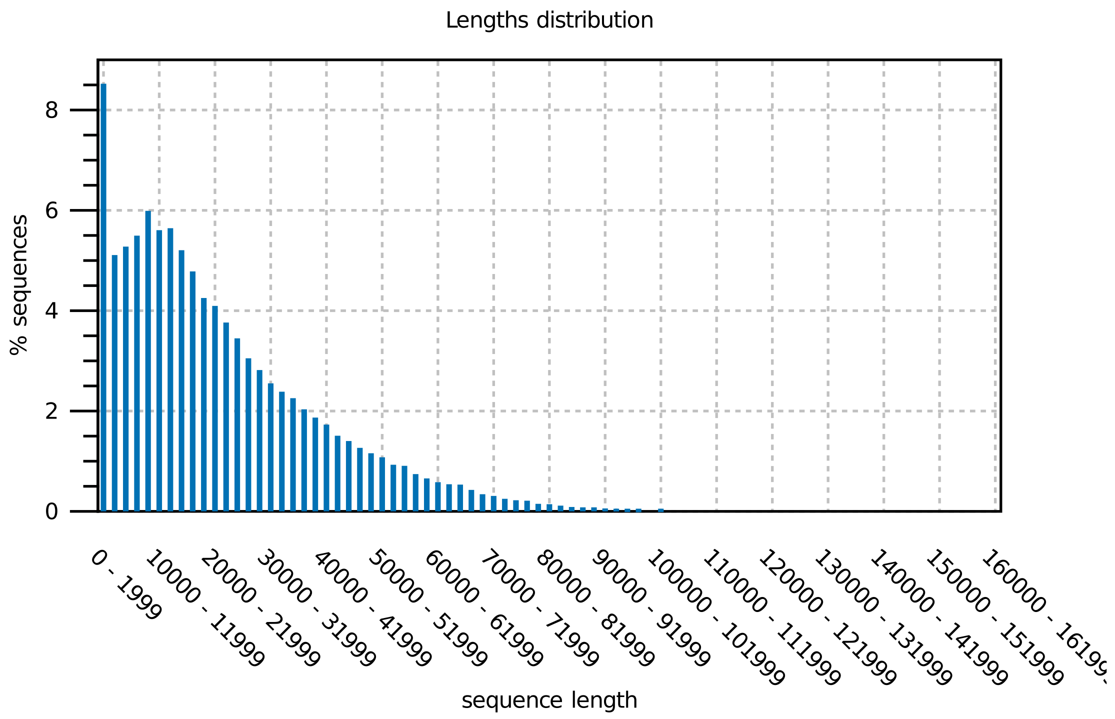

Distribution of sequence lengths. In cases of untrimmed Illumina reads it will just contain a single peak.
x: sequence length in base-pairs
y: number of sequences featuring a particular length normalized to the total number of sequences

2.2 GC-content

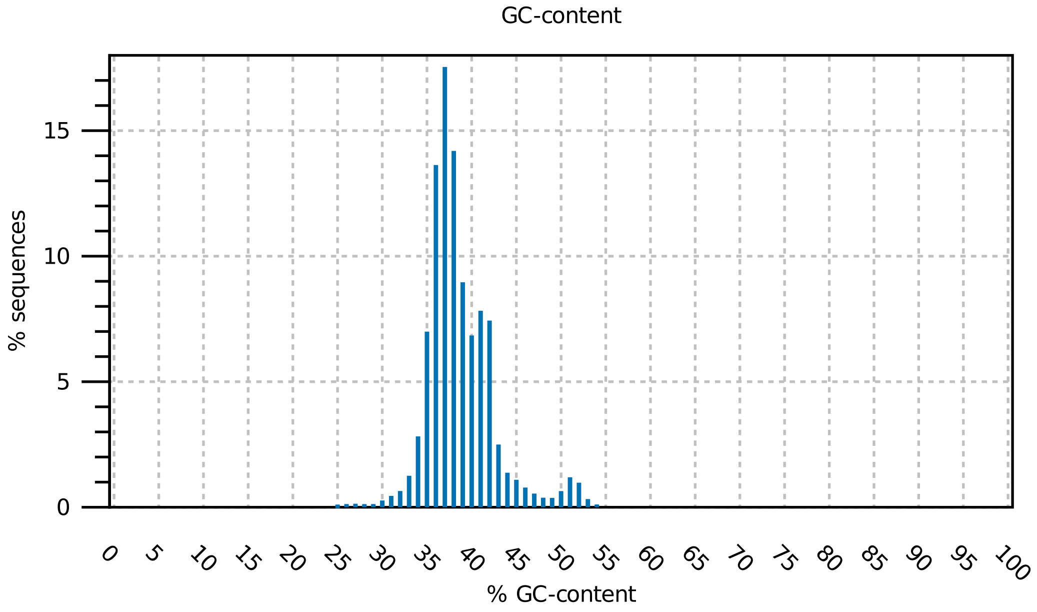

Distribution of GC-contents. The GC-content of a sequence is calculated as the number of GC-bases compared to all bases (including ambiguous bases).
x: relative GC-content of a sequence in percent
y: number of sequences featuring particular GC-percentages normalized to the total number of sequences

2.3 Ambiguous base-content

No ambiguous bases detected

2.4 Quality distribution

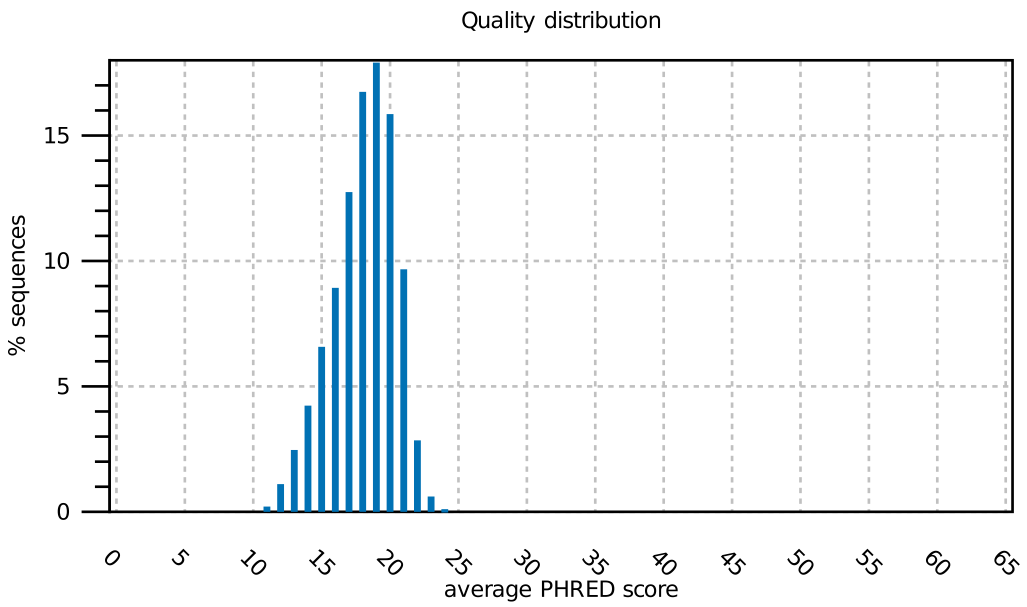

Distribution of average sequence quality scores. The quality of a sequence is calculated as the arithmetic mean of its base qualities.
x: PHRED-score
y: number of sequences observed at that qual. score normalized to the total number of sequences

3. Per-base analysis

The plots in this section only show 153369 bases as the coverage for the remaining bases is less than 0.005%.

3.1 Coverage

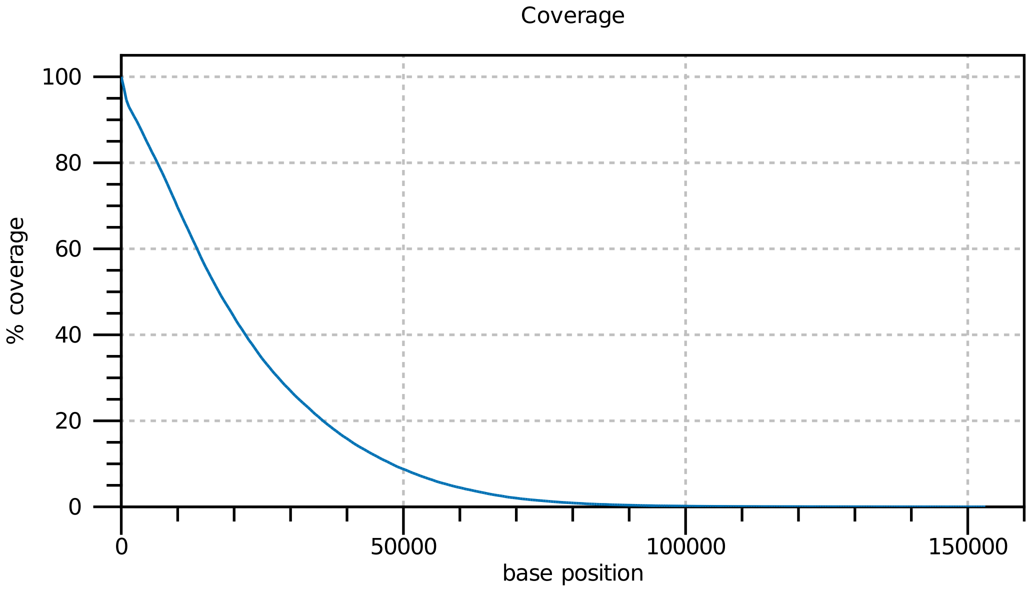

The number of sequences that support (cover) the individual base positions. In cases of untrimmed Illumina reads it will just contain a line.
x: base position
y: number of sequences covering individual base positions normalized to the total number of sequences

3.2 Nucleotide contributions

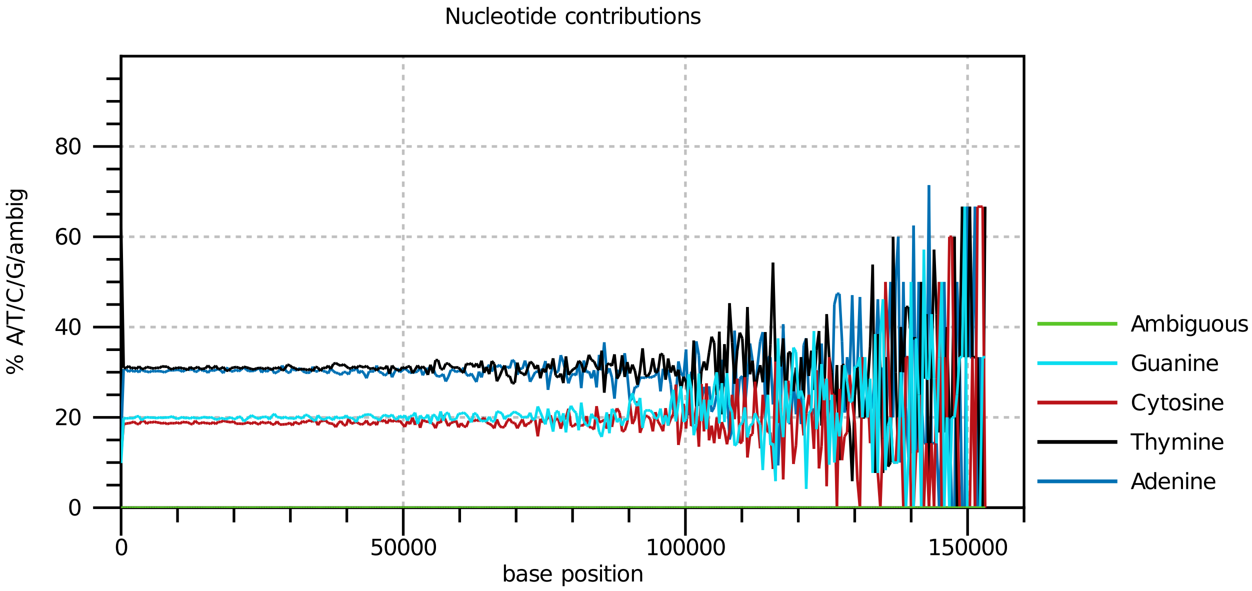

Coverages for the four DNA nucleotides and ambiguous bases.
x: base position

y: number of nucleotides observed per type normalized to the total number of nucleotides observed at that position

3.3 GC-content

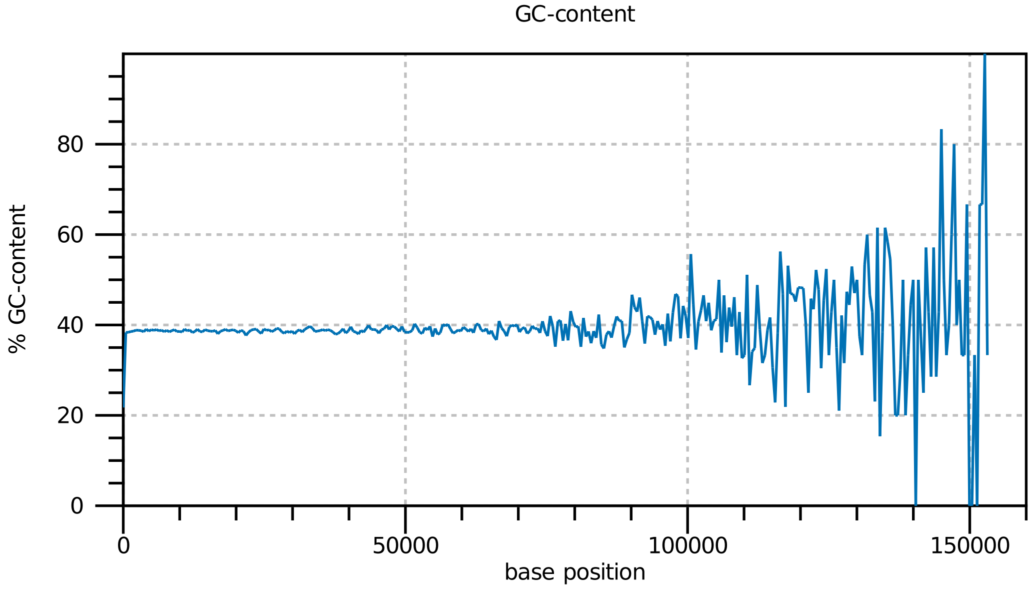

Combined coverage of G- and C-bases.
x: base position
y: number of G- and C-bases observed at current position normalized to the total number of bases observed at that position

3.4 Ambiguous base-content

No ambiguous bases detected

3.5 Quality distribution

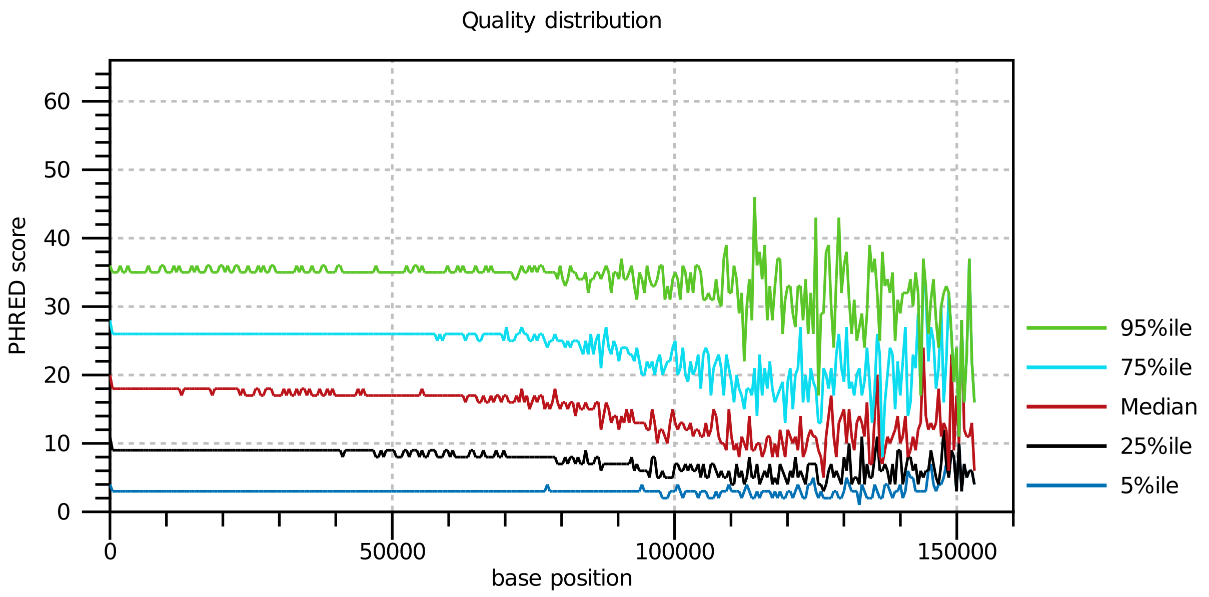

Base-quality distribution along the base positions.
x: base position
y: median & percentiles of quality scores observed at that base position

4. Over-representation analyses

The enchriched 5-mers plot in this section only shows 153369 bases as the coverage for the remaining bases is less than 0.005%.

4.1 Enriched 5-mers

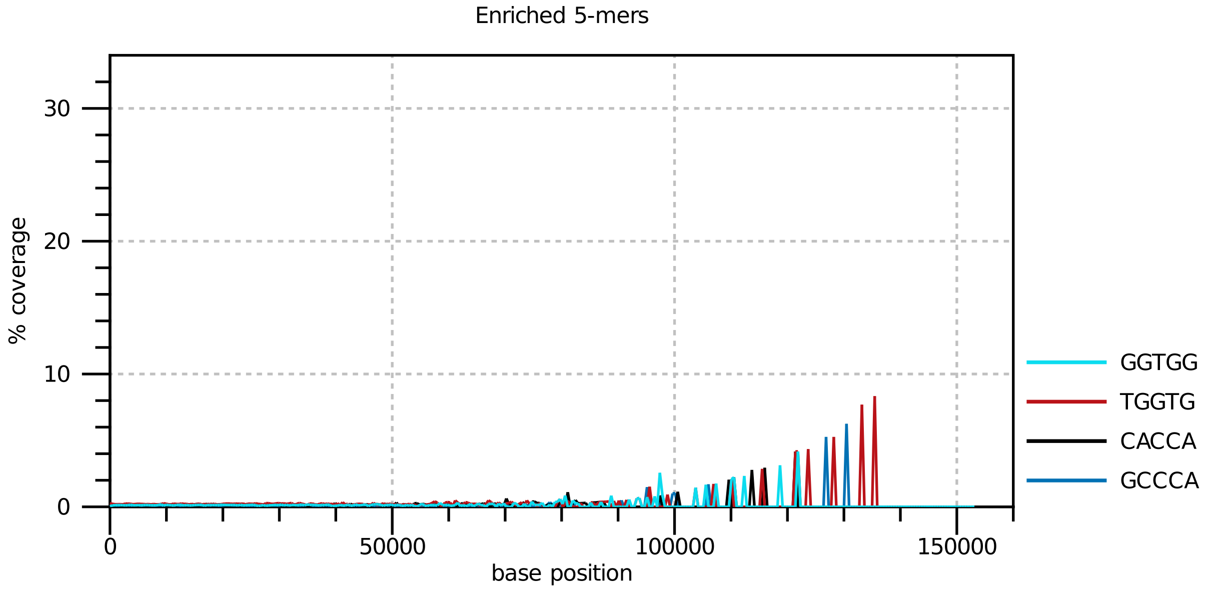

The five most-overrepresented 5-mers. The over-representation of a 5-mer is calculated as the ratio of the observed and expected 5-mer frequency. The expected frequency is calculated as product of the empirical nucleotide probabilities that make up the 5-mer. (5-mers that contain ambiguous bases are ignored)
x: base position
y: number of times a 5-mer has been observed normalized to all 5-mers observed at that position

4.2 Sequence duplication levels

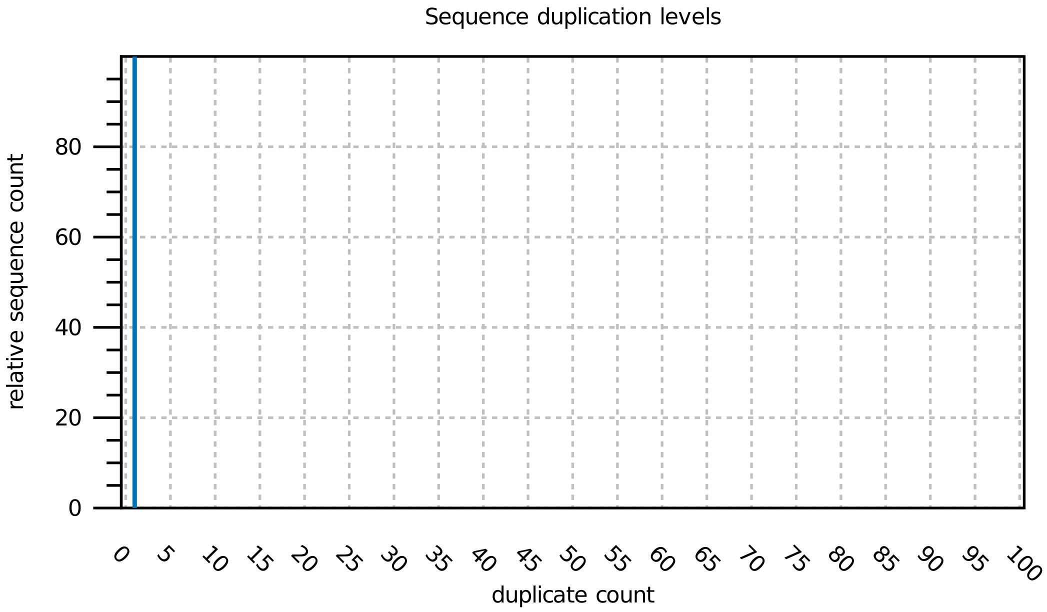

Duplication level distribution. Duplication levels are simply the count of how often a particular sequence has been found.
x: duplicate count
y: number of sequences that have been found that many times normalized to the number of unique sequences

4.3 Duplicated sequences

A table of over-represented sequences is given in the supplementary report

Sequencing QC Report
Based upon: 40,421 sequences in 1 data set
Generated by: root
Creation date: Mon Nov 22 10:01:13 CET 2021
Software: CLC Genomics Workbench 21.0.4

Table of contents

1. Summary 3
2. Per-sequence analysis 3
    2.1 Lengths distribution 3
    2.2 GC-content 4
    2.3 Ambiguous base-content 4
    2.4 Quality distribution 5
3. Per-base analysis 5
    3.1 Coverage 6
    3.2 Nucleotide contributions 6
    3.3 GC-content 7
    3.4 Ambiguous base-content 7
    3.5 Quality distribution 8
4. Over-representation analyses 8
    4.1 Enriched 5-mers 9
    4.2 Sequence duplication levels 9
    4.3 Duplicated sequences 10

1. Summary

Creation date:	Mon Nov 22 10:01:13 CET 2021
Generated by:	root
Software:	CLC Genomics Workbench 21.0.4
Based upon:	1 data set
Cherry_2-2:	40,421 sequences
Total sequences in data set	40,421 sequences
Total nucleotides in data set	918,137,204 nucleotides

2. Per-sequence analysis

2.1 Lengths distribution

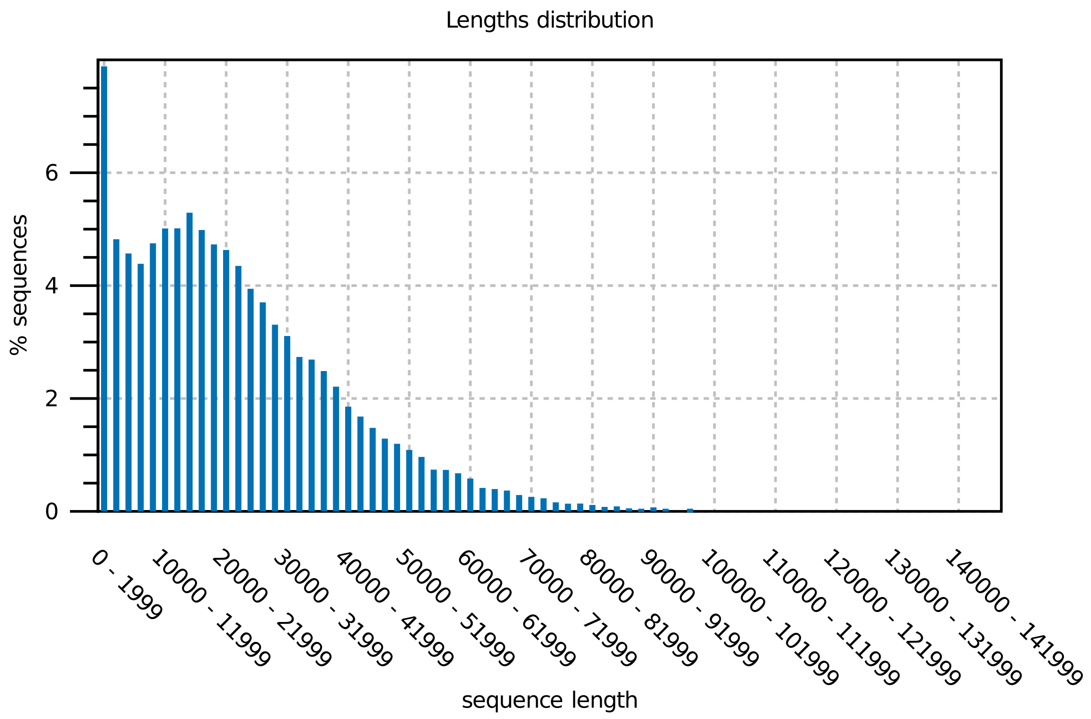

Distribution of sequence lengths. In cases of untrimmed Illumina reads it will just contain a single peak.
x: sequence length in base-pairs
y: number of sequences featuring a particular length normalized to the total number of sequences

2.2 GC-content

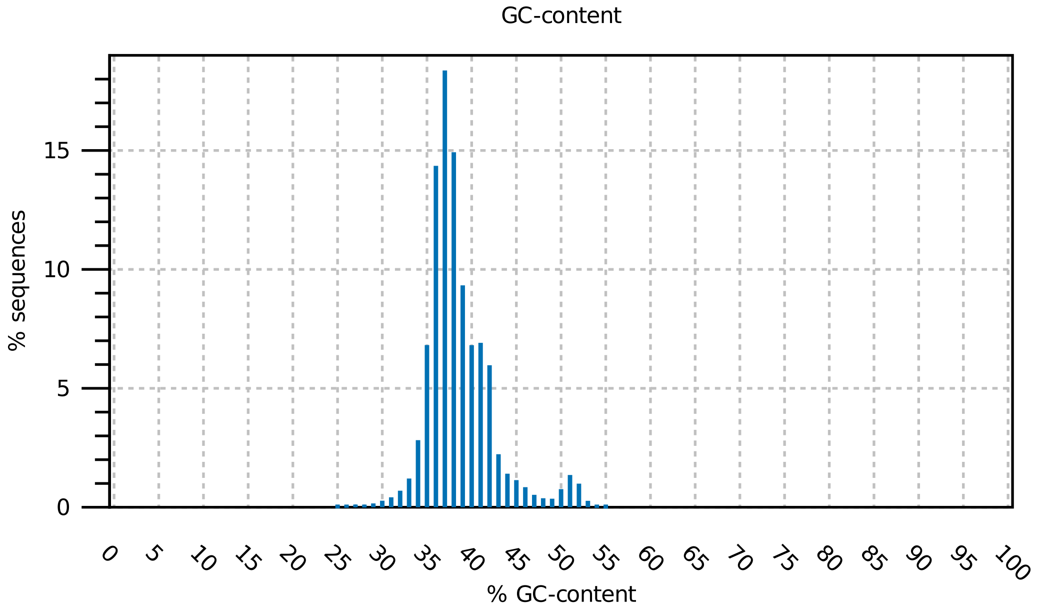

Distribution of GC-contents. The GC-content of a sequence is calculated as the number of GC-bases compared to all bases (including ambiguous bases).
x: relative GC-content of a sequence in percent
y: number of sequences featuring particular GC-percentages normalized to the total number of sequences

2.3 Ambiguous base-content

No ambiguous bases detected

2.4 Quality distribution

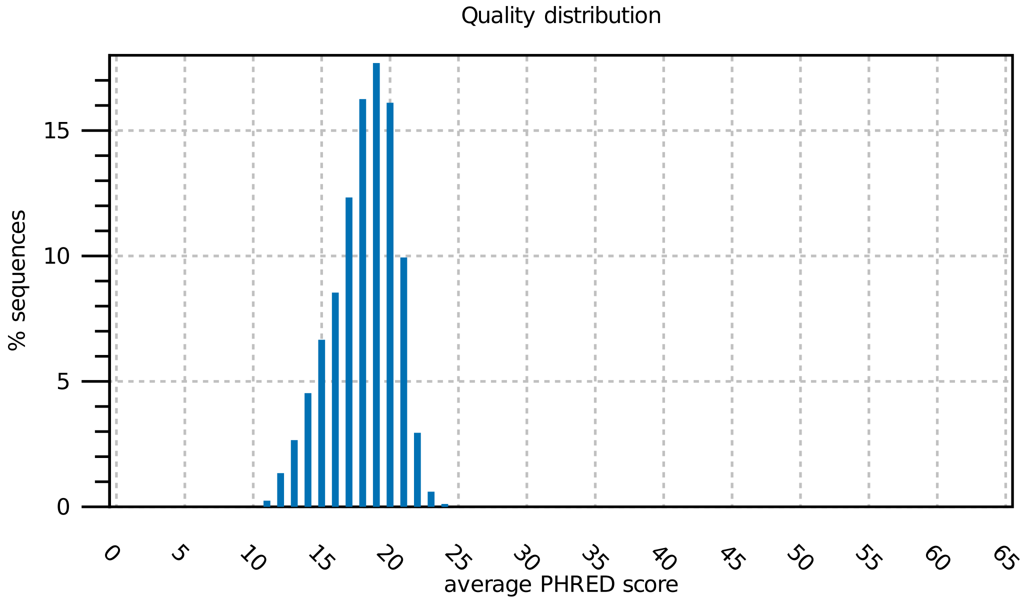

Distribution of average sequence quality scores. The quality of a sequence is calculated as the arithmetic mean of its base qualities.
x: PHRED-score
y: number of sequences observed at that qual. score normalized to the total number of sequences

3. Per-base analysis

The plots in this section only show 132530 bases as the coverage for the remaining bases is less than 0.005%.

3.1 Coverage

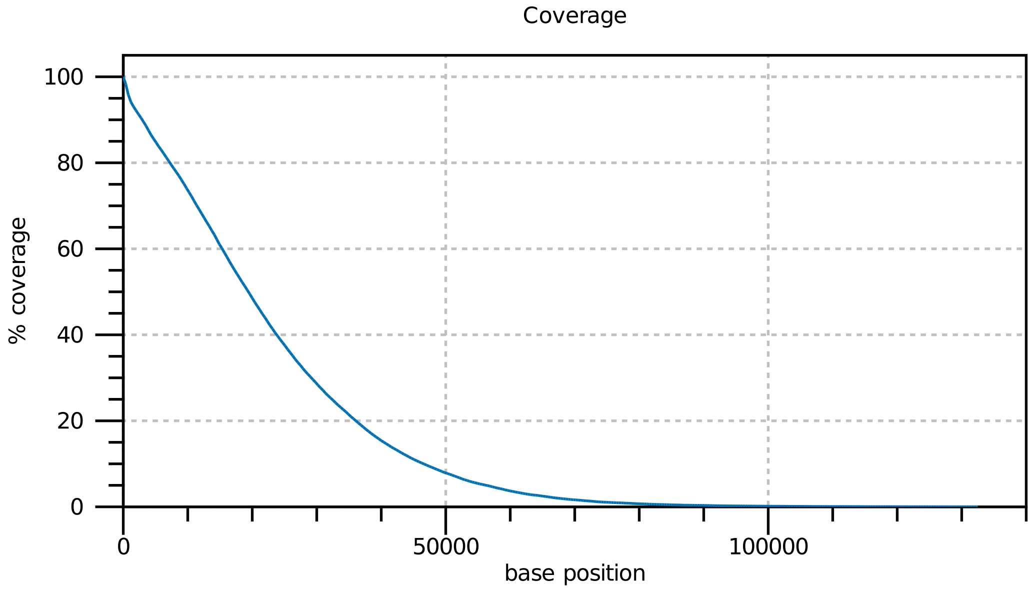

The number of sequences that support (cover) the individual base positions. In cases of untrimmed Illumina reads it will just contain a line.
x: base position
y: number of sequences covering individual base positions normalized to the total number of sequences

3.2 Nucleotide contributions

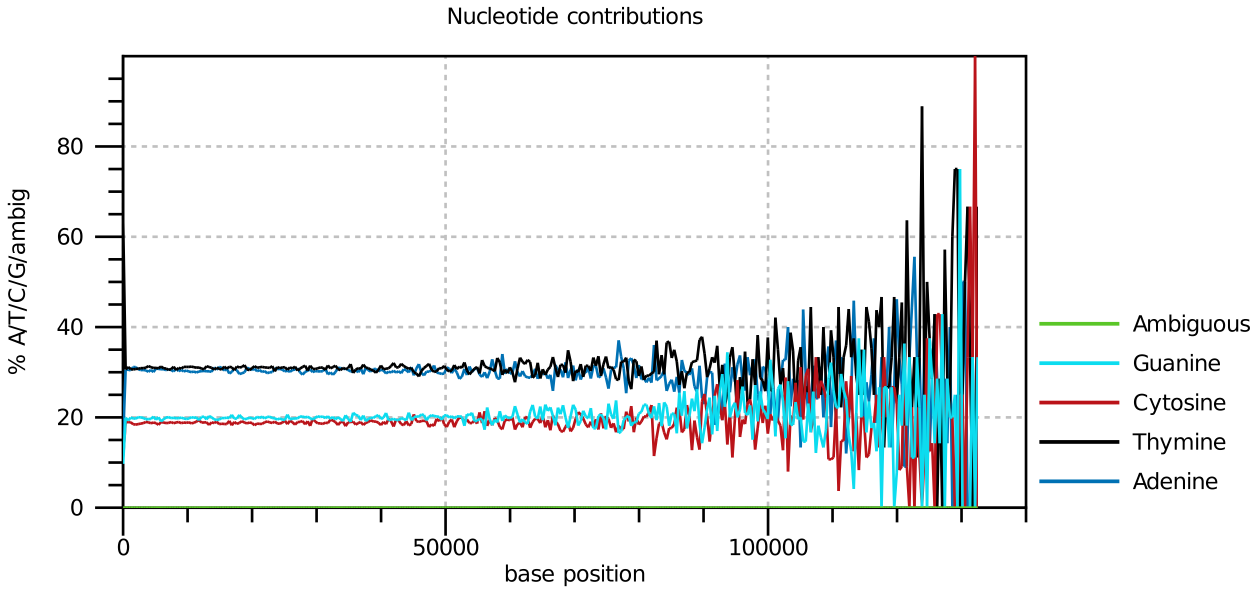

Coverages for the four DNA nucleotides and ambiguous bases.
x: base position

y: number of nucleotides observed per type normalized to the total number of nucleotides observed at that position

3.3 GC-content

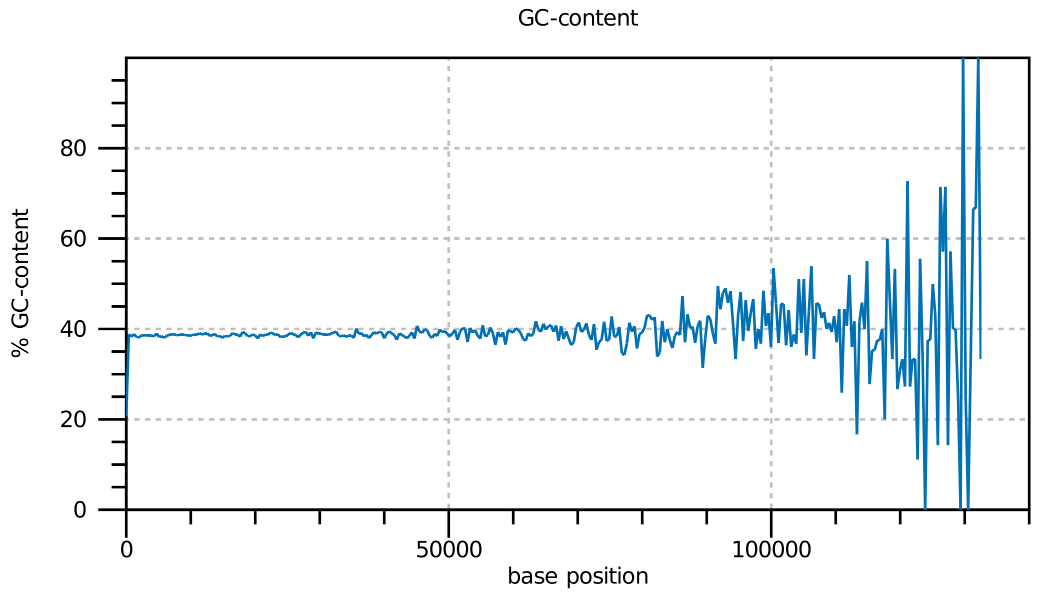

Combined coverage of G- and C-bases.
x: base position
y: number of G- and C-bases observed at current position normalized to the total number of bases observed at that position

3.4 Ambiguous base-content

No ambiguous bases detected

3.5 Quality distribution

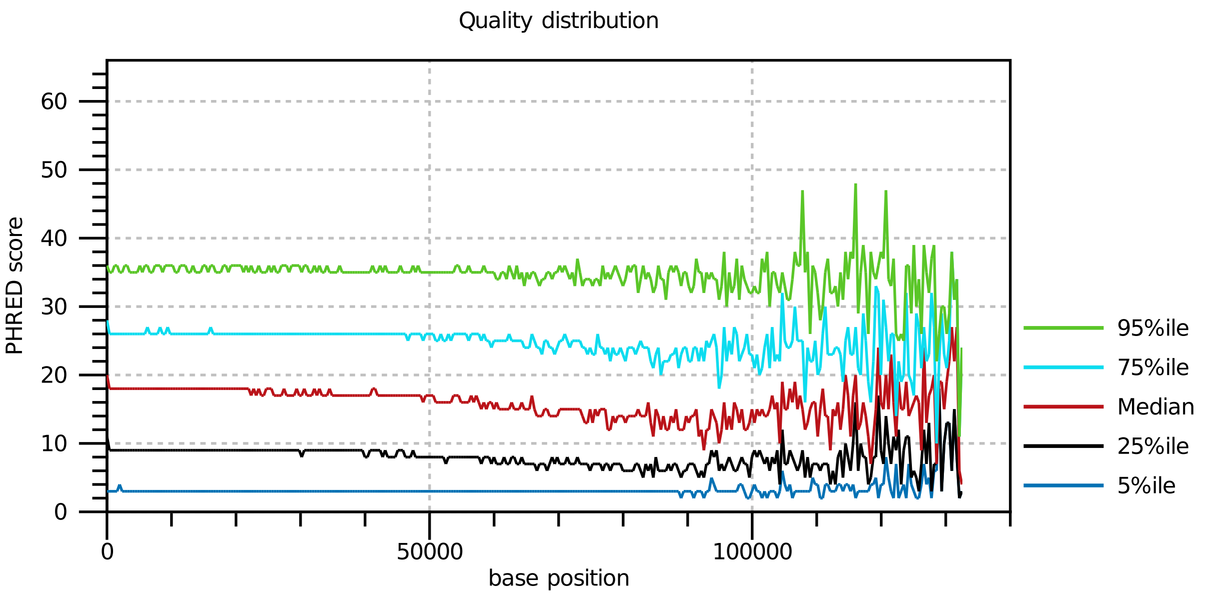

Base-quality distribution along the base positions.
x: base position
y: median & percentiles of quality scores observed at that base position

4. Over-representation analyses

The enchriched 5-mers plot in this section only shows 132530 bases as the coverage for the remaining bases is less than 0.005%.

4.1 Enriched 5-mers

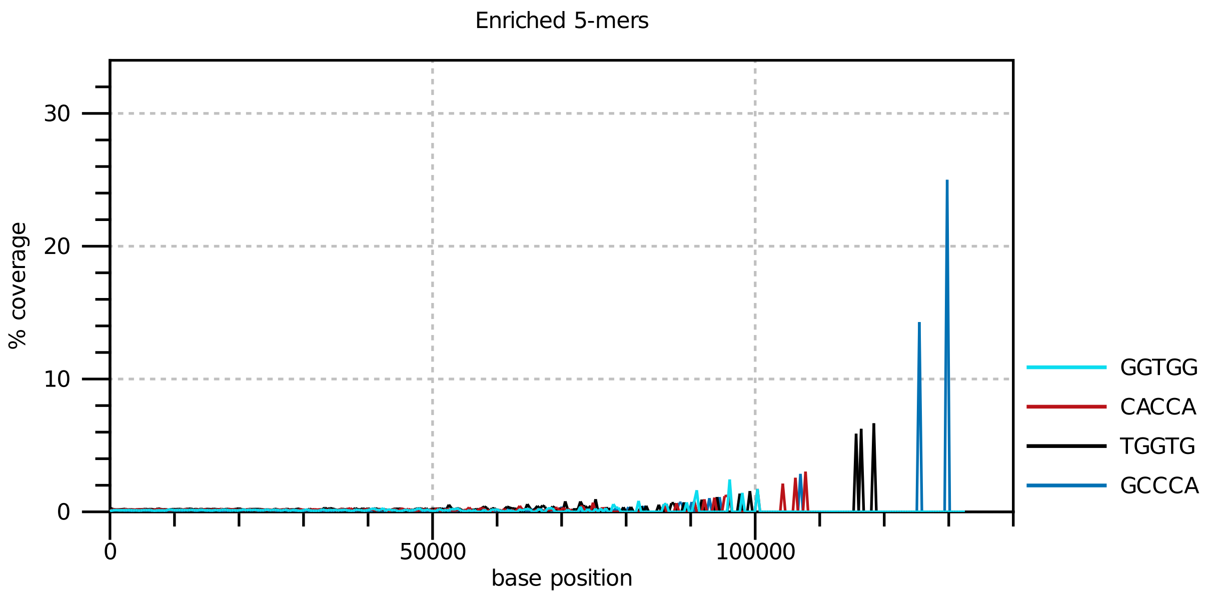

The five most-overrepresented 5-mers. The over-representation of a 5-mer is calculated as the ratio of the observed and expected 5-mer frequency. The expected frequency is calculated as product of the empirical nucleotide probabilities that make up the 5-mer. (5-mers that contain ambiguous bases are ignored)
x: base position
y: number of times a 5-mer has been observed normalized to all 5-mers observed at that position

4.2 Sequence duplication levels

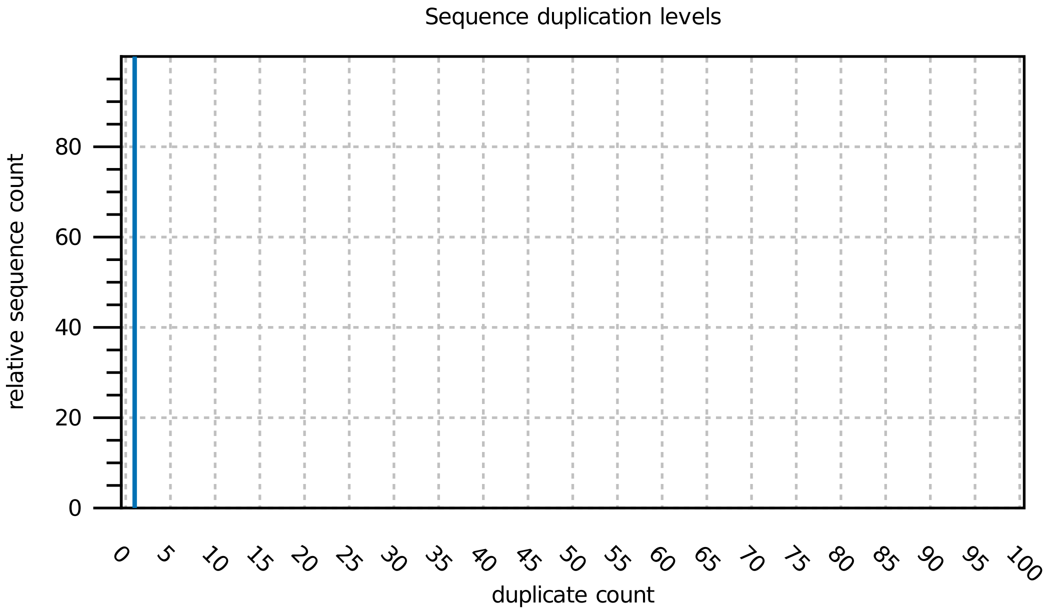

Duplication level distribution. Duplication levels are simply the count of how often a particular sequence has been found.
x: duplicate count
y: number of sequences that have been found that many times normalized to the number of unique sequences

4.3 Duplicated sequences

A table of over-represented sequences is given in the supplementary report

Sequencing QC Report
Based upon: 143,470 sequences in 1 data set
Generated by: root
Creation date: Mon Nov 22 10:13:35 CET 2021
Software: CLC Genomics Workbench 21.0.4

Table of contents

1. Summary 3
2. Per-sequence analysis 3
    2.1 Lengths distribution 3
    2.2 GC-content 4
    2.3 Ambiguous base-content 4
    2.4 Quality distribution 5
3. Per-base analysis 5
    3.1 Coverage 6
    3.2 Nucleotide contributions 6
    3.3 GC-content 7
    3.4 Ambiguous base-content 7
    3.5 Quality distribution 8
4. Over-representation analyses 8
    4.1 Enriched 5-mers 9
    4.2 Sequence duplication levels 9
    4.3 Duplicated sequences 10

1. Summary

Creation date:	Mon Nov 22 10:13:35 CET 2021
Generated by:	root
Software:	CLC Genomics Workbench 21.0.4
Based upon:	1 data set
Cherry_3-1:	143,470 sequences
Total sequences in data set	143,470 sequences
Total nucleotides in data set	3,362,441,292 nucleotides

2. Per-sequence analysis

2.1 Lengths distribution

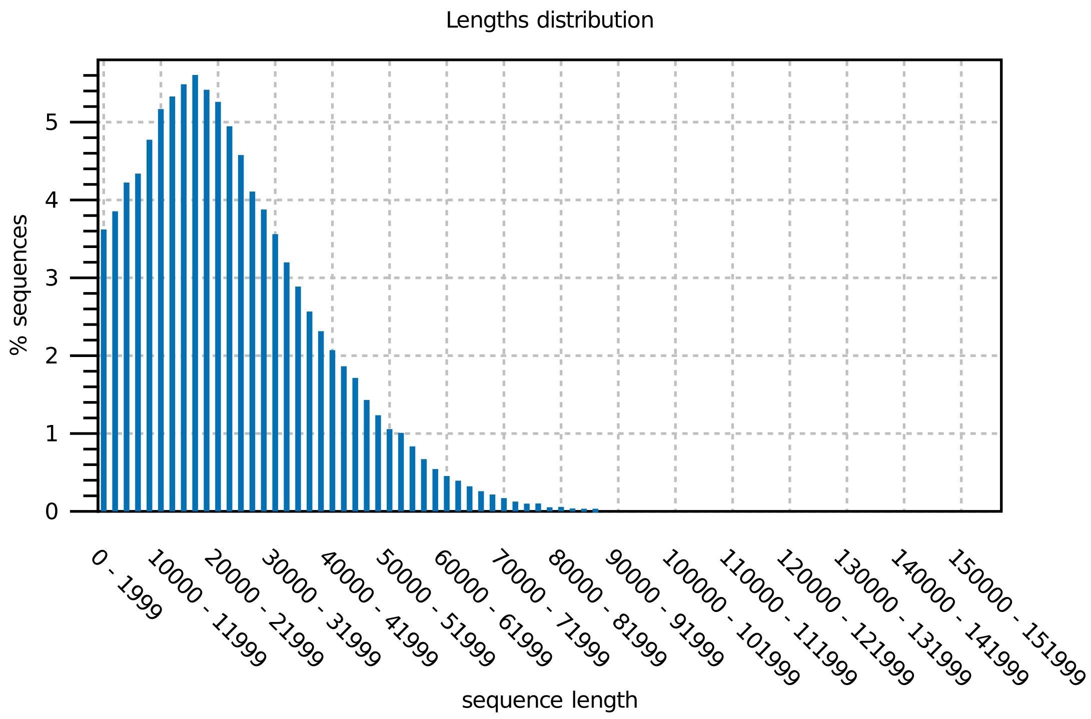

Distribution of sequence lengths. In cases of untrimmed Illumina reads it will just contain a single peak.
x: sequence length in base-pairs
y: number of sequences featuring a particular length normalized to the total number of sequences

2.2 GC-content

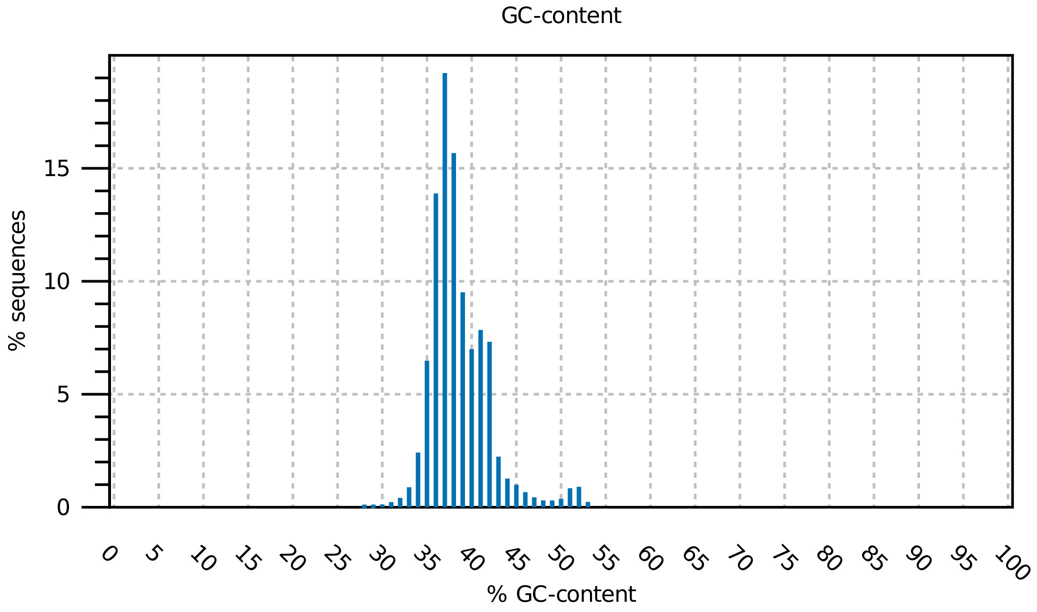

Distribution of GC-contents. The GC-content of a sequence is calculated as the number of GC-bases compared to all bases (including ambiguous bases).
x: relative GC-content of a sequence in percent
y: number of sequences featuring particular GC-percentages normalized to the total number of sequences

2.3 Ambiguous base-content

No ambiguous bases detected

2.4 Quality distribution

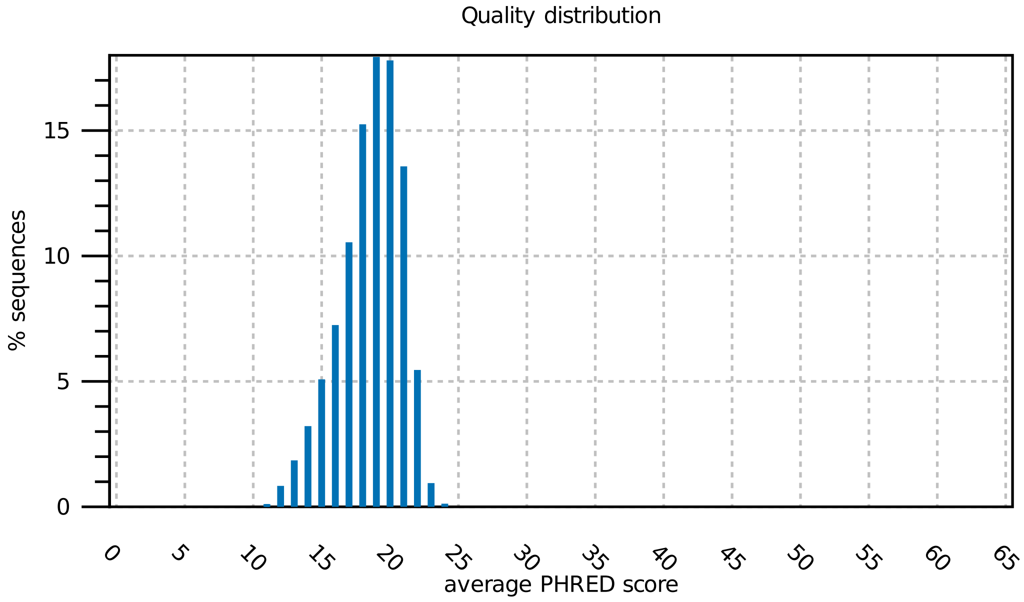

Distribution of average sequence quality scores. The quality of a sequence is calculated as the arithmetic mean of its base qualities.
x: PHRED-score
y: number of sequences observed at that qual. score normalized to the total number of sequences

3. Per-base analysis

The plots in this section only show 132451 bases as the coverage for the remaining bases is less than 0.005%.

3.1 Coverage

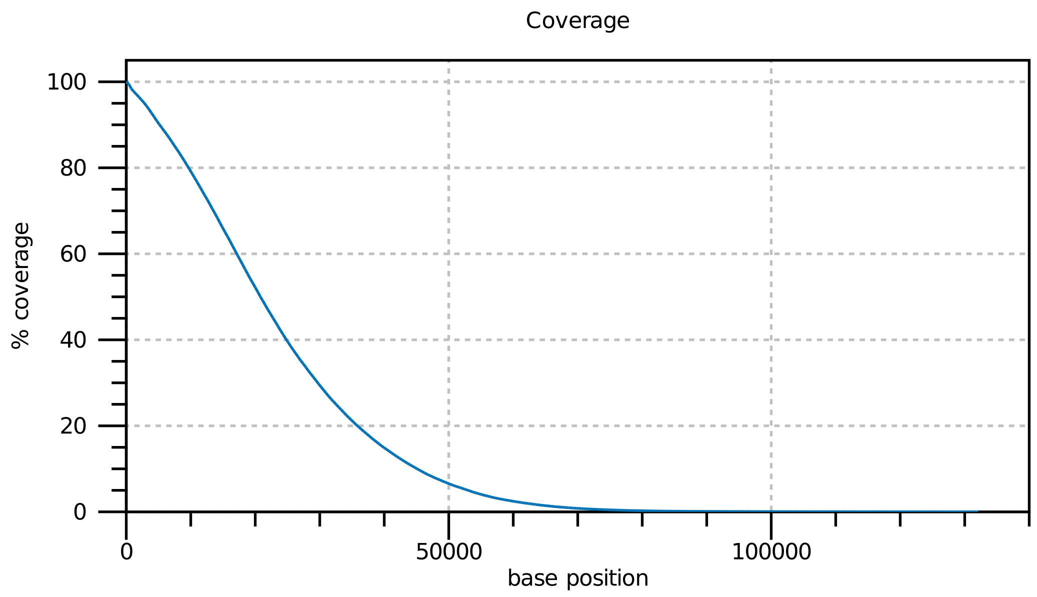

The number of sequences that support (cover) the individual base positions. In cases of untrimmed Illumina reads it will just contain a line.
x: base position
y: number of sequences covering individual base positions normalized to the total number of sequences

3.2 Nucleotide contributions

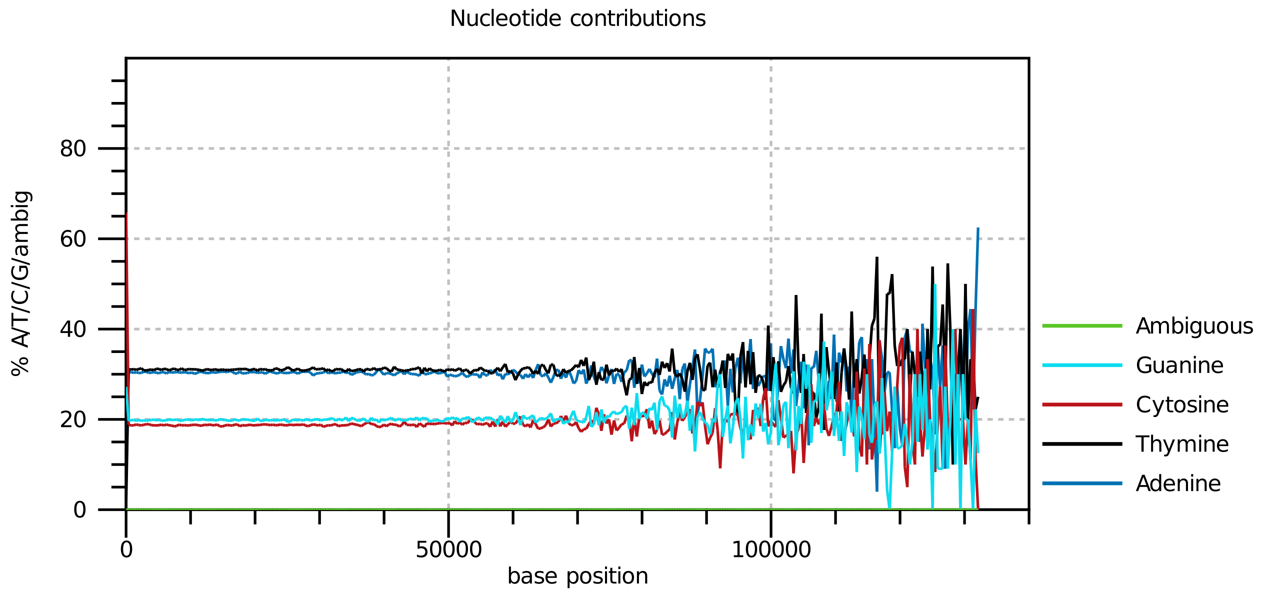

Coverages for the four DNA nucleotides and ambiguous bases.
x: base position

y: number of nucleotides observed per type normalized to the total number of nucleotides observed at that position

3.3 GC-content

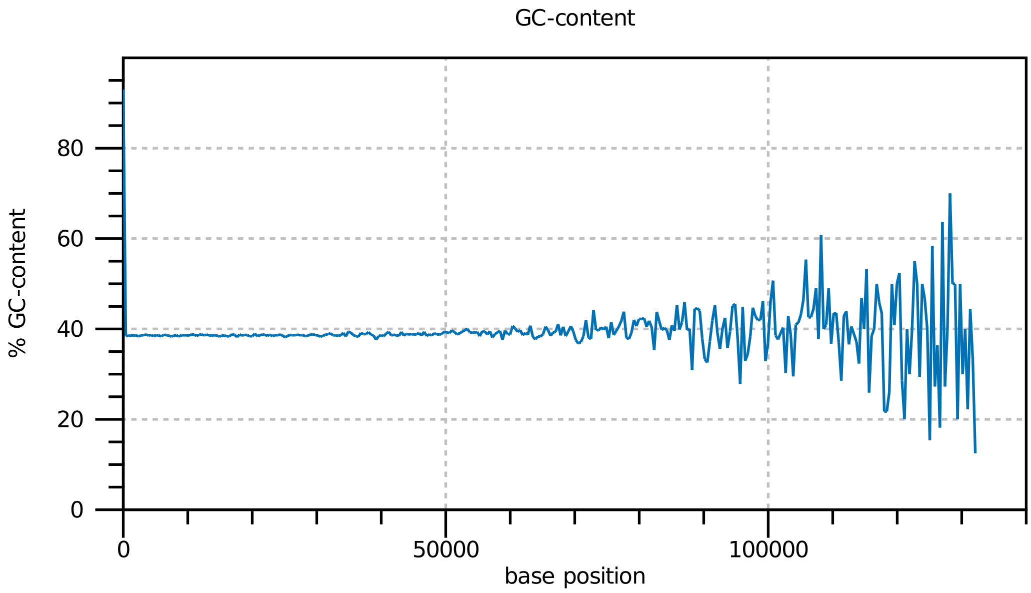

Combined coverage of G- and C-bases.
x: base position
y: number of G- and C-bases observed at current position normalized to the total number of bases observed at that position

3.4 Ambiguous base-content

No ambiguous bases detected

3.5 Quality distribution

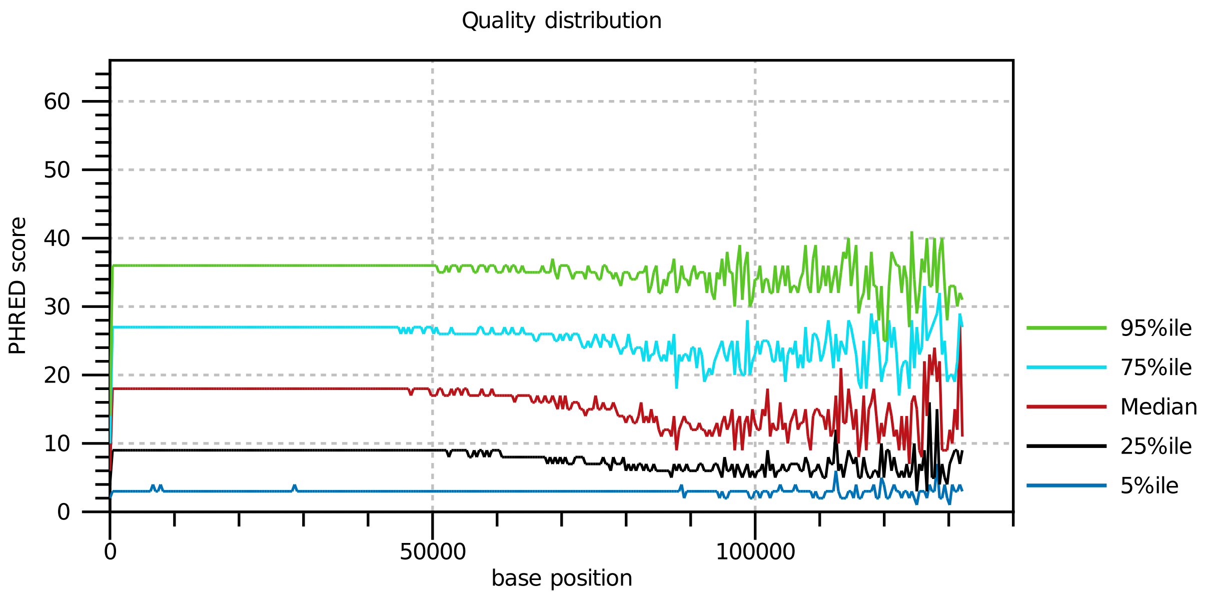

Base-quality distribution along the base positions.
x: base position
y: median & percentiles of quality scores observed at that base position

4. Over-representation analyses

The enchriched 5-mers plot in this section only shows 132451 bases as the coverage for the remaining bases is less than 0.005%.

4.1 Enriched 5-mers

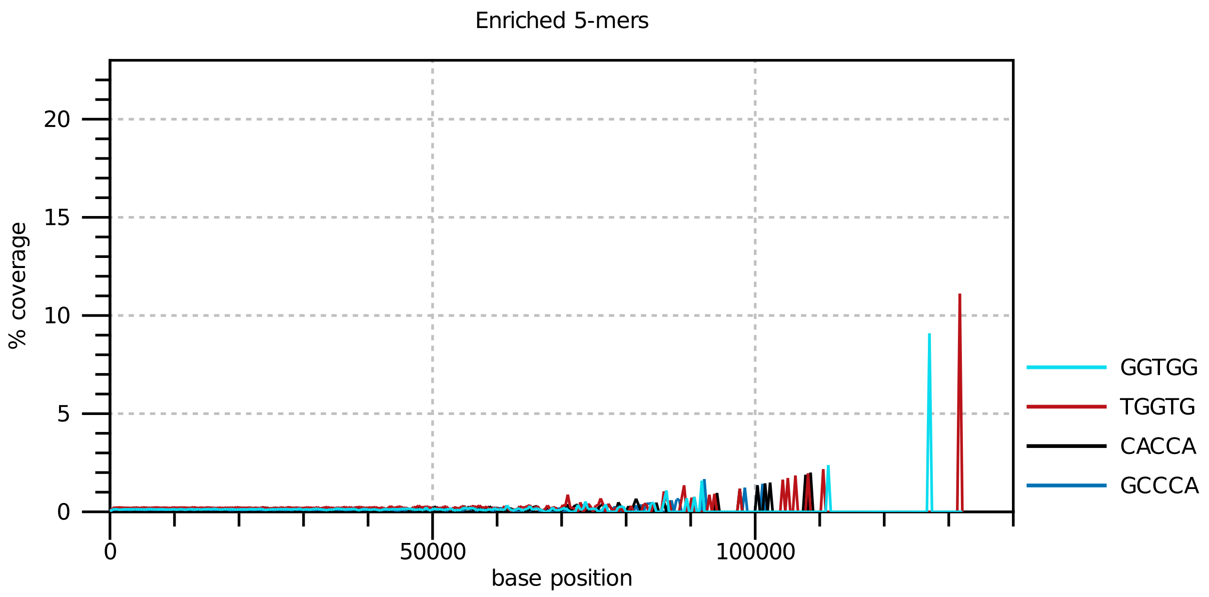

The five most-overrepresented 5-mers. The over-representation of a 5-mer is calculated as the ratio of the observed and expected 5-mer frequency. The expected frequency is calculated as product of the empirical nucleotide probabilities that make up the 5-mer. (5-mers that contain ambiguous bases are ignored)
x: base position
y: number of times a 5-mer has been observed normalized to all 5-mers observed at that position

4.2 Sequence duplication levels

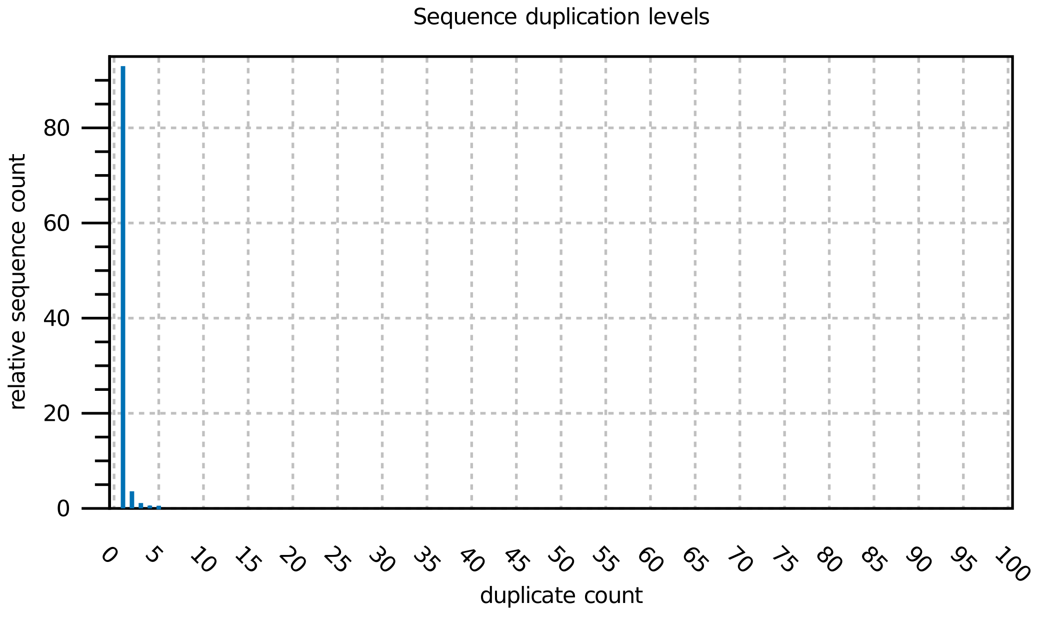

Duplication level distribution. Duplication levels are simply the count of how often a particular sequence has been found.
x: duplicate count
y: number of sequences that have been found that many times normalized to the number of unique sequences

4.3 Duplicated sequences

A table of over-represented sequences is given in the supplementary report

Sequencing QC Report
Based upon: 109,846 sequences in 1 data set
Generated by: root
Creation date: Mon Nov 22 10:24:32 CET 2021
Software: CLC Genomics Workbench 21.0.4

Table of contents

1. Summary 3
2. Per-sequence analysis 3
    2.1 Lengths distribution 3
    2.2 GC-content 4
    2.3 Ambiguous base-content 4
    2.4 Quality distribution 5
3. Per-base analysis 5
    3.1 Coverage 6
    3.2 Nucleotide contributions 6
    3.3 GC-content 7
    3.4 Ambiguous base-content 7
    3.5 Quality distribution 8
4. Over-representation analyses 8
    4.1 Enriched 5-mers 9
    4.2 Sequence duplication levels 9
    4.3 Duplicated sequences 10

1. Summary

Creation date:	Mon Nov 22 10:24:32 CET 2021
Generated by:	root
Software:	CLC Genomics Workbench 21.0.4
Based upon:	1 data set
Cherry_3-14:	109,846 sequences
Total sequences in data set	109,846 sequences
Total nucleotides in data set	2,563,398,969 nucleotides

2. Per-sequence analysis

2.1 Lengths distribution

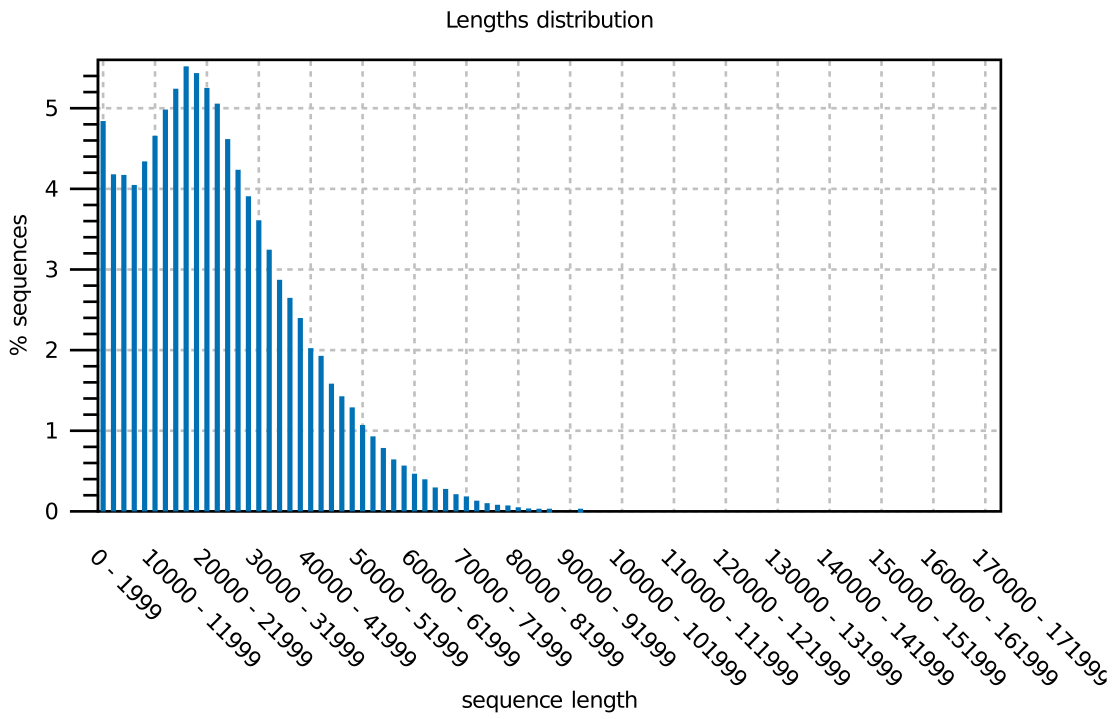

Distribution of sequence lengths. In cases of untrimmed Illumina reads it will just contain a single peak.
x: sequence length in base-pairs
y: number of sequences featuring a particular length normalized to the total number of sequences

2.2 GC-content

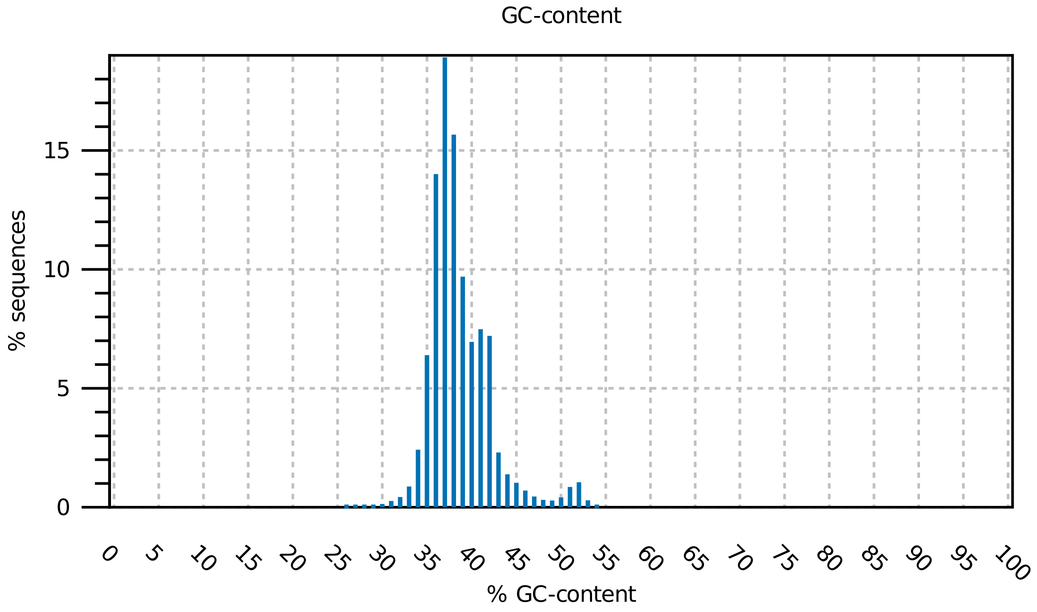

Distribution of GC-contents. The GC-content of a sequence is calculated as the number of GC-bases compared to all bases (including ambiguous bases).
x: relative GC-content of a sequence in percent
y: number of sequences featuring particular GC-percentages normalized to the total number of sequences

2.3 Ambiguous base-content

No ambiguous bases detected

2.4 Quality distribution

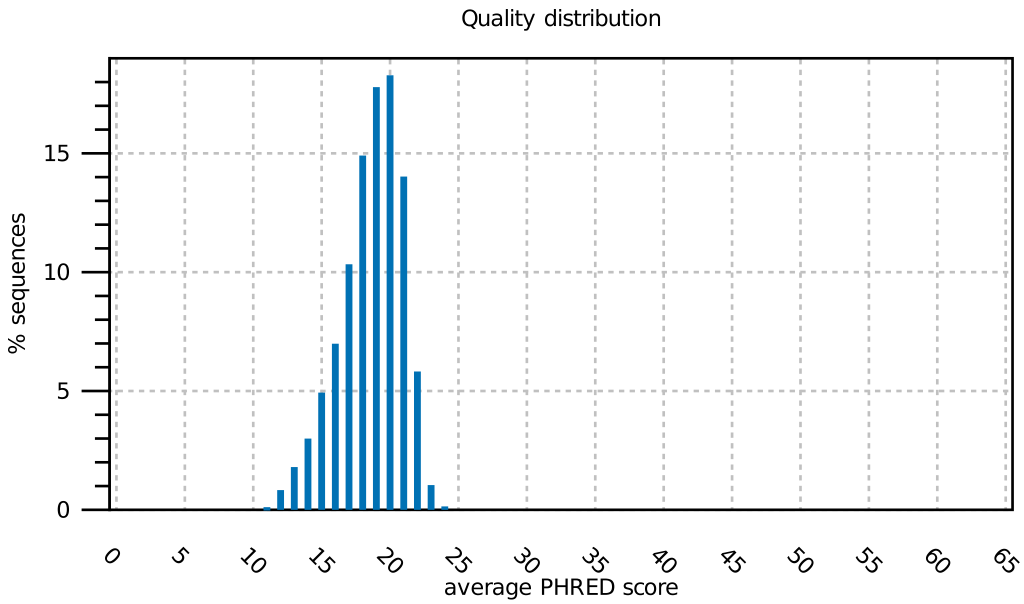

Distribution of average sequence quality scores. The quality of a sequence is calculated as the arithmetic mean of its base qualities.
x: PHRED-score
y: number of sequences observed at that qual. score normalized to the total number of sequences

3. Per-base analysis

The plots in this section only show 127488 bases as the coverage for the remaining bases is less than 0.005%.

3.1 Coverage

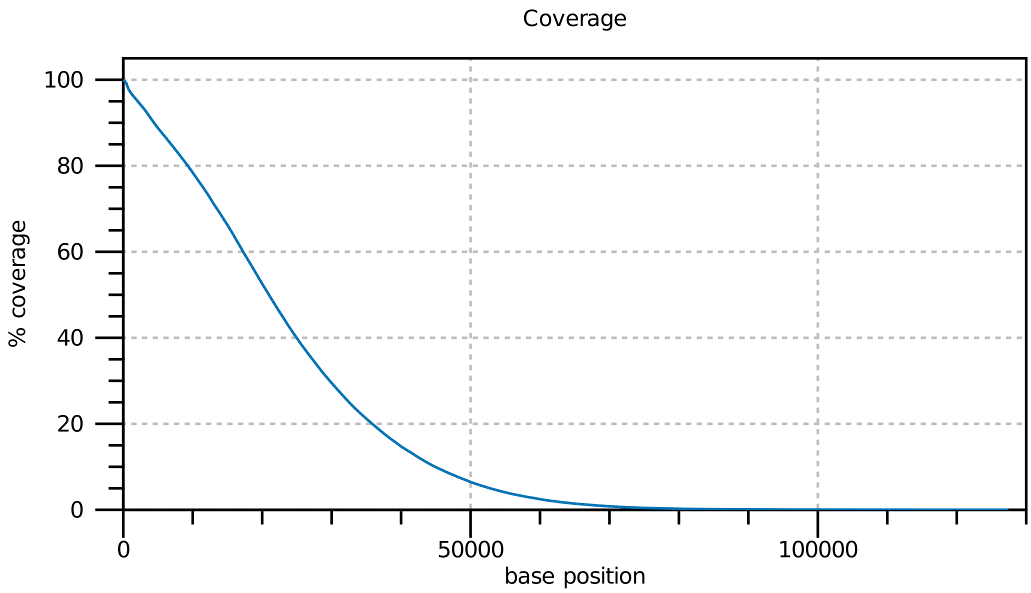

The number of sequences that support (cover) the individual base positions. In cases of untrimmed Illumina reads it will just contain a line.
x: base position
y: number of sequences covering individual base positions normalized to the total number of sequences

3.2 Nucleotide contributions

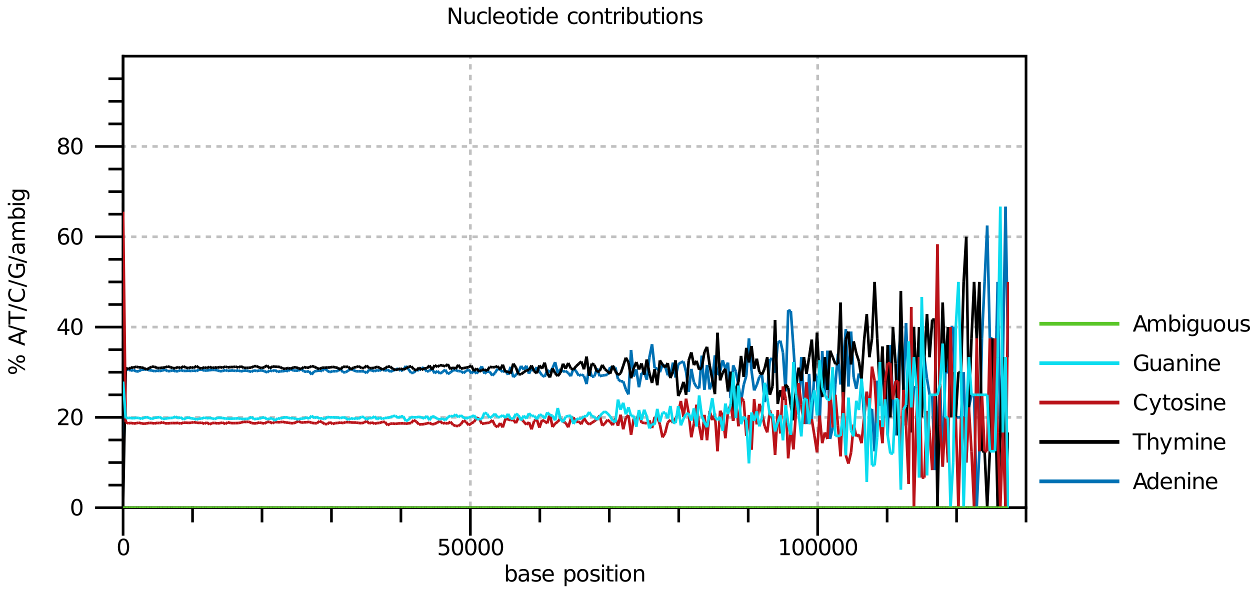

Coverages for the four DNA nucleotides and ambiguous bases.
x: base position

y: number of nucleotides observed per type normalized to the total number of nucleotides observed at that position

3.3 GC-content

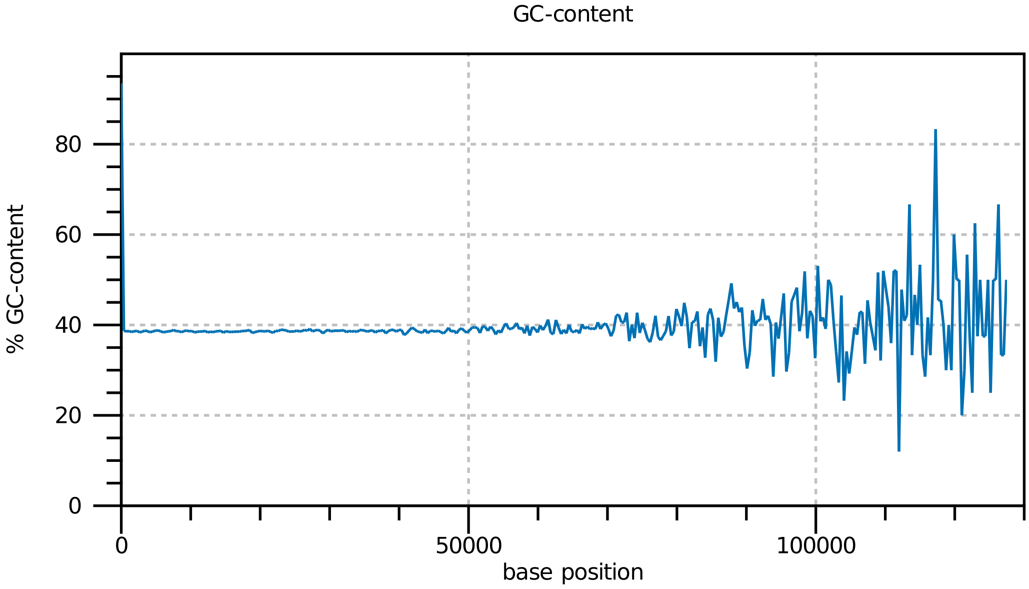

Combined coverage of G- and C-bases.
x: base position
y: number of G- and C-bases observed at current position normalized to the total number of bases observed at that position

3.4 Ambiguous base-content

No ambiguous bases detected

3.5 Quality distribution

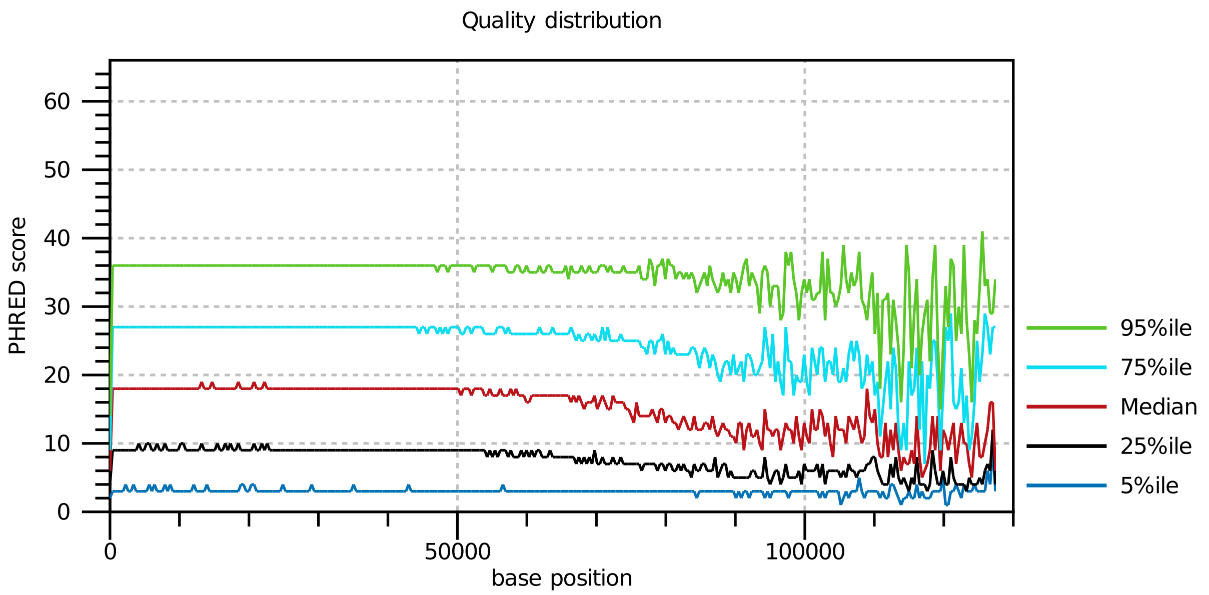

Base-quality distribution along the base positions.
x: base position
y: median & percentiles of quality scores observed at that base position

4. Over-representation analyses

The enchriched 5-mers plot in this section only shows 127488 bases as the coverage for the remaining bases is less than 0.005%.

4.1 Enriched 5-mers

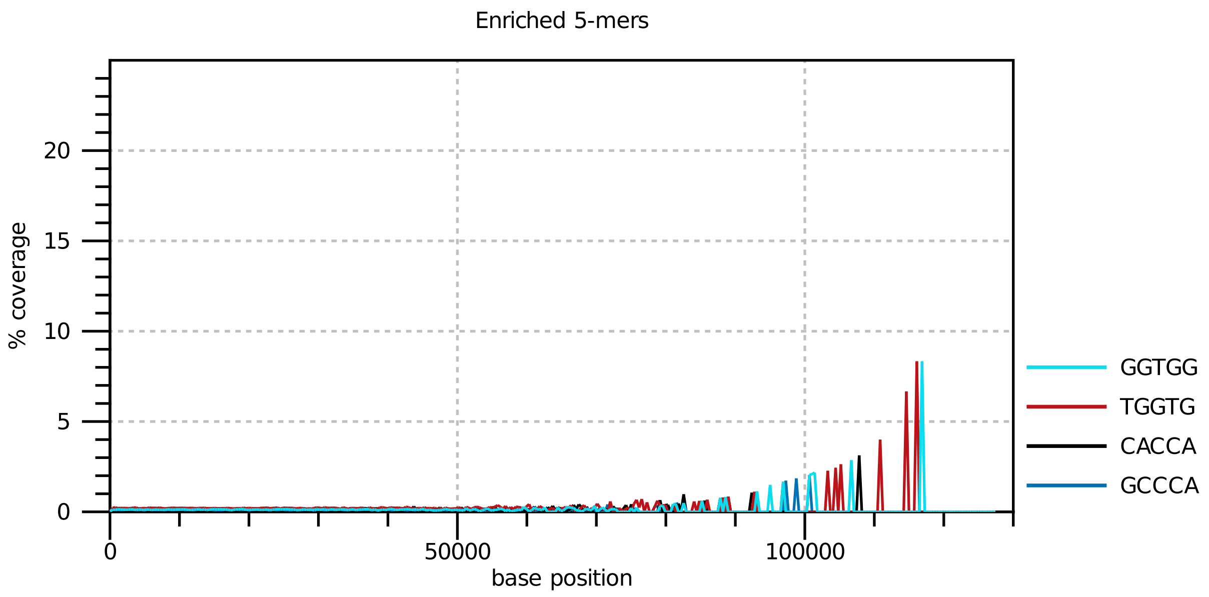

The five most-overrepresented 5-mers. The over-representation of a 5-mer is calculated as the ratio of the observed and expected 5-mer frequency. The expected frequency is calculated as product of the empirical nucleotide probabilities that make up the 5-mer. (5-mers that contain ambiguous bases are ignored)
x: base position
y: number of times a 5-mer has been observed normalized to all 5-mers observed at that position

4.2 Sequence duplication levels

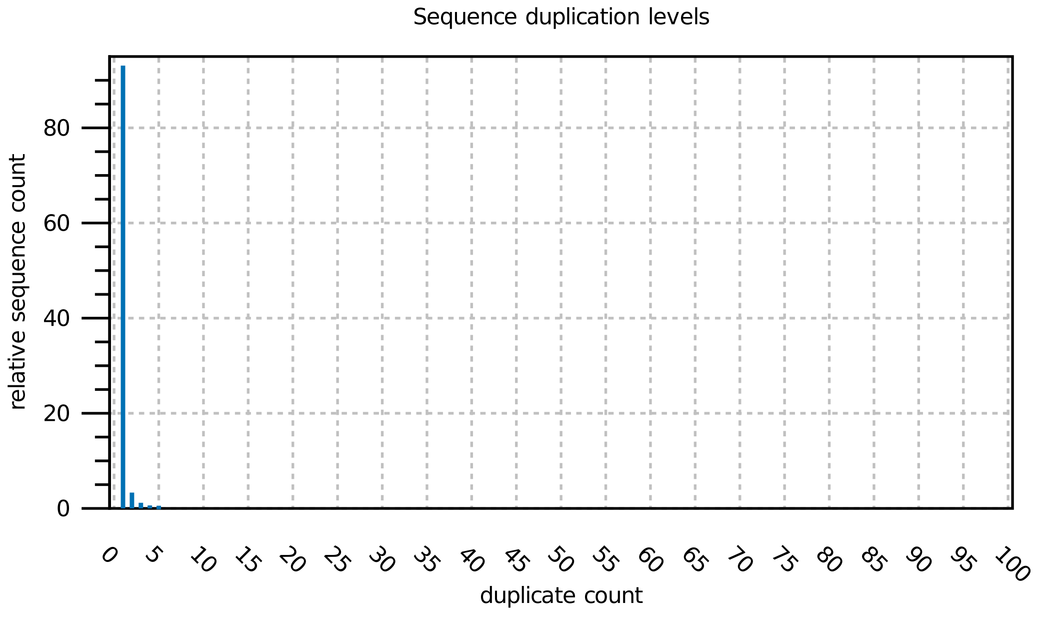

Duplication level distribution. Duplication levels are simply the count of how often a particular sequence has been found.
x: duplicate count
y: number of sequences that have been found that many times normalized to the number of unique sequences

4.3 Duplicated sequences

A table of over-represented sequences is given in the supplementary report
